# The Kids Obesity Prevention Program: Cluster Randomized Controlled Trial to Evaluate a Serious Game for the Prevention and Treatment of Childhood Obesity

**DOI:** 10.2196/15725

**Published:** 2020-04-24

**Authors:** Isabelle Mack, Nadine Reiband, Carolin Etges, Sabrina Eichhorn, Norbert Schaeffeler, Guido Zurstiege, Caterina Gawrilow, Katja Weimer, Riyad Peeraully, Martin Teufel, Gunnar Blumenstock, Katrin Elisabeth Giel, Florian Junne, Stephan Zipfel

**Affiliations:** 1 Department of Psychosomatic Medicine and Psychotherapy University Medical Hospital Tübingen Germany; 2 Department of School Psychology University of Tübingen Tübingen Germany; 3 Department of Media Studies Tübingen University of Tübingen Tübingen Germany; 4 Department of Psychosomatic Medicine and Psychotherapy Ulm University Medical Center Ulm Germany; 5 Department of Paediatric Surgery Nottingham Children's Hospital Nottingham United Kingdom; 6 LVR-Clinic for Psychosomatic Medicine and Psychotherapy University of Duisburg-Essen Essen Germany; 7 Institute for Clinical Epidemiology and Applied Biometry University of Tübingen Tübingen Germany

**Keywords:** children, serious game, nutrition, stress, energy density

## Abstract

**Background:**

Health games provide opportunities for the treatment and prevention of childhood obesity. We developed a motion-controlled serious game for children that addresses 3 core topics of nutrition, physical activity, and stress coping. It is the first serious game that extensively targets the dietary energy density principle (DED-P) in relation to nutrition. The game is intended to provide an additional educational component for the prevention and treatment of obesity in children.

**Objective:**

The Kids Obesity Prevention study aimed to evaluate the newly developed game and to evaluate how well children are able to understand and apply the DED-P.

**Methods:**

This cluster randomized controlled trial collected data from 82 primary school children aged 9 to 12 years and their parents at baseline (T0), at 2 weeks after study commencement (T1), and at the 4-week follow-up (T2). The dropout rate was 3.6%. The intervention group (IG) played the game within 2 weeks (2 sessions with different game modules). One part of the game involves selection of food with the lower energy density when presented with a pair of foods. This allows assessment of whether the children have understood the DED-P and whether they can apply it to unknown foods under time pressure. The control group (CG) received a brochure about the food pyramid concept and physical activity. The primary outcome was the gain in knowledge (nutrition and stress coping) and measured with a pretested questionnaire. The secondary outcomes were the maintenance of knowledge, application of the DED-P, feelings during game play, game acceptance, and behavioral measures (physical activity, media consumption, and dietary intake).

**Results:**

The knowledge score ranging from 0 to 100 increased from T0 (IG: 53 [SD 10], CG: 50 [SD 11]) to T1 (IG: 69 [SD 11], CG: 52 [SD 12]) in IG versus CG (*P*<.001). At T2, the knowledge score of IG remained at the same level as that of T1. Game data showed that after DED-P education, the classification under time pressure of unknown versus known food pairs according to their DED category was similar (hit rate around 70%). Overall, 95% of the children liked the game very much or much. No group changes were observed at the behavioral level.

**Conclusions:**

The Kids Obesity Prevention program sustainably increased knowledge in the areas of nutrition and stress coping, and children were able to apply the DED-P.

**Trial Registration:**

ClinicalTrials.gov NCT02551978; https://clinicaltrials.gov/ct2/show/NCT02551978

## Introduction

### Obesity and Weight Management Principles

Obesity and its associated comorbidities such as type 2 diabetes and hypertension are a severe public health problem. Unfortunately, many school-aged children are already overweight or obese [1], and most of them will remain overweight and obese in adulthood [2]. Obese children and adolescents often differ in several physiological [3-6] and psychological parameters [6-8] when compared with their peers. Some children even suffer from comorbidities [6] and are victims of stigmatization [9,10]. Therefore, it is imperative to establish effective weight gain prevention [11] and treatment programs [12] during childhood.

Weight management principles are aimed at reducing energy intake by improving diet and eating behavior and at increasing energy expenditure by increasing physical activity and avoiding sedentary behaviors [13]. At the dietary level, portion size and the dietary energy density (DED) of foods [13,14] are critical factors. Knowledge of appropriate food portion sizes is important in view of the increase in portion sizes over recent decades [15]. In adults, a recent short-term study reported that it is possible to recalibrate the perception of food portion sizes. In this study, participants were served small food portions, which affected their perceptions of what constitutes a normal-sized serving and resulted in choosing to eat less food in the future [16]. The food pyramid or my plate concept has been applied for many years to guide portion sizes for the different food groups [17,18], although the flexibility of adapting to personal preferences is limited when using this concept.

Obese adults, adolescents, and children have increased gastric capacities and might need to eat larger portions to perceive satiety in comparison with normal-weight participants, which is problematic if foods high in DED are consumed [19-21]. Adults eat consistent food amounts over several days even if the DED is varied, and this may also be true for children aged 3 to 5 years [14]. Thus, consideration of the DED of foods may be an important factor for weight regulation.

The success of lifestyle interventions is critically dependent on psychosocial and psychological aspects, such as stress [22]. Learning how to cope with stress may be an important factor in weight regulation, as stress can be associated with an unfavorable lifestyle, including low physical activity and high energy intake by consuming more foods with high DED and probably fewer foods with low DED [23]. For interventions to be successful in childhood, family support and goal commitment are also necessary [22].

### Digital Media

Digital media offer a ubiquitous and highly accepted tool, especially in younger age groups. In the United Kingdom, for instance, 3- to 7-year-old children watch TV for about 2 hours a day, whereas 8- to 15-year-old children spend 2.5 hours in the Web, watch TV for 2 hours a day, and play games for 2 hours a day [24]. Similar results have been reported in Germany [25]. Although screen time is considered a risk factor for childhood obesity [26], there appear to be differences between passive and active screen time, with active being less problematic [27]. For lifestyle interventions, the well-considered and appropriate use of digital media offers enormous advantages [28-30]. These digital media include serious games, which are games designed to fulfill a *serious* purpose by providing education from health professionals via a digital device [31]. Serious games can be applied to convey health-related information and can also use interactive components to reinforce the information and train behaviors, for example, by incorporating cognitive bias therapy into games [32,33].

In a recent systematic review, the abilities and limitations of video games, including exergames (focusing on increasing activity) and serious games, to combat and prevent childhood obesity were investigated. It was found that the children like playing the games, and on a qualitative level, most studies report positive effects on obesity-related outcomes (improvement of weight-related parameters, physical activity, or dietary behavior and knowledge). However, at the quantitative level, the observed effects were small [29]. When analyzing the teaching of concepts regarding nutrition and eating habits by games, most studies focus on the concepts of food pyramid and nutritional balance [34-43], fruit-vegetable or 5-a-day (aimed at increasing fruit and vegetables to 5 portions per day [44-47]), or the Mediterranean diet [48]. Intake of dietary fat, excess sugar [42,49], liquids, packed snacks, and fast food [39,47,48] have been addressed in other studies. None of the games investigated have addressed the DED-P in detail or considered psychosocial or psychological aspects of nutrition and eating habits, such as stress coping [29].

### Objective and Hypothesis

We developed a motion-controlled serious game for children, the Kids Obesity Prevention (KOP) program, addressing the areas of nutrition, physical activity, and stress coping. In addition to a motion control interface, a tablet is used for knowledge-based and cognitive tasks. Unlike in most other games, the nutritional episodes in the game presented here relate to the nutrition pyramid and, beyond that, to the DED of food and drink, as well as stress and stress-coping strategies, such as relaxation exercises. The game was developed to be applied in obesity prevention and as an additional educational component of inpatient and outpatient settings for obesity treatment in children.

The aim of the KOP study was to evaluate the game in a cluster randomized controlled trial with two parallel groups in a primary school setting in children aged 9 to 12 years. The primary outcome was the gain in knowledge about important lifestyle factors with the focus on nutrition, especially the DED-P. The secondary outcomes were the maintenance of knowledge, the application of the DED-P, game acceptance, dietary habits, physical activity, and media consumption. Our primary hypothesis was that after intervention, (1) the intervention group (IG) would have higher knowledge scores compared with the control group (CG). We further hypothesized that (2) after the 4-week follow-up, the knowledge scores of IG would be higher than that at baseline but lower than the scores immediately after game play; (3) children would understand and apply the DED-P; (4) children would enjoy playing the game, and game acceptance would be high; and (5) no changes at the behavioral level would be observed because of the primary study aim and design.

## Methods

### Study Design and Participants

The KOP study was a cluster randomized controlled trial involving school children aged 9 to 12 years; it was conducted from September to November 2015 in Germany. The study period was 2 weeks, and the follow-up was conducted 4 weeks after study termination. The inclusion criterion was an age between 9 and 12 years (all children who were in the fourth grade of primary school). The exclusion criterion was children with major linguistic difficulties.

At baseline, participants were assessed for knowledge about a healthy lifestyle, with a focus on nutrition issues, weight and height, physical activity, and dietary behavior. Parents provided information about their child regarding physical activity, dietary behavior, and media consumption.

The detailed study procedure for the intervention program was set up before participant enrollment in arrangement with the teachers. The recruitment of children took place at a parents’ evening of the school classes. Parents and children provided written informed consent. The study protocol was approved by the ethics committee of the medical faculty of the Eberhard Karls University Tübingen and the University Hospital Tübingen, Germany (050/2014BO1). The KOP-1 study was registered before the study commencement at ClinicalTrials.gov (NCT02551978).

### Randomization and Masking

Five fourth grade classes in a single school were randomly allocated to an IG or a CG. To ensure balanced age and sex distributions and to avoid interactions between children in different groups within one class, classes were randomized en bloc to one of the groups by a coin toss. The randomization took place before study commencement. Owing to the nature of the intervention, participants and outcome assessors could not be blinded to treatment assignments.

### Treatments

#### Intervention

The IG played the game twice over a 2-week period, with a different selection of game modules played at each of the two sessions (Section Game Modules and Multimedia Appendix 1). Each session took about 45 min. An investigator was present during the whole time. The game [50] starts with a framing story of a child living in a medieval fantasy world presented by a storyteller showing pictures in an ancient book on a screen. The story is about the competition between two villages to regain knowledge about nutrition and a healthy lifestyle. Competitive elements of the story and elements where children had supportive function of the character aimed at motivating the children to engage in the game. After the presentation of the story in a video, the player has to move an avatar in a 3D medieval world to walk from the site of one task to another. The player’s physical movement is necessary to make the avatar move in the game. Therefore, the player walks on the spot, lifting the knees upward (gaming PC: ARLT Silent Gamer GTX 650 [Windows 7], Intel Core i3 3240 [2× 3.4 GHz], 8 GB RAM, 2000 GB hard disk drive, NVIDIA GeForce GTX 650; Kamera Xbox One Microsoft Kinect Sensor; and BenQ MW851UST DLP-Beamer). When the avatar reaches certain locations for tasks to be completed, a switch to touch screens occurs (Acer Iconia Tab W510, NT.L0KEG.001). Depending on the gamer, the locations are reached within 1 to 2 min. The topics addressed by the game belong to the categories of nutrition, physical activity, and stress coping. The nutrition segment of the game deals with the food pyramid and the sugar content of liquids, and focuses on factors that are important for satiety, therefore teaching the concept of DED-P extensively. A self-reflective diagnostic tool to analyze daily food intake is also offered. Other aspects including eustress, distress, and stress-coping strategies are addressed by relaxation exercises and a tool for reflecting and planning everyday activities. An overview and description of the game modules is given below in the section Game Modules, screenshots are provided as Multimedia Appendix 2, and a video is provided as Multimedia Appendix 3.

#### Control Condition

The CG received basic information about a healthy lifestyle via a brochure from the informational service of the Federal Ministry of Food and Agriculture entitled “So macht Essen Spaß (How to make food fun)” and was handed out to the children at the beginning of the study phase. The CG received the intervention of the IG after study completion because of ethical considerations (the ethics committee wished the CG to play the game too).

#### Game Modules

##### Pack Your Backpack With Food

Both game sessions start with this self-reflective diagnostic tool to analyze the individual daily food intake. In this task, the children must pack as many foods into a backpack as they think they need to consume in a normal day to be energy balanced. Before the children start the game, the sex, age, height, weight, and activity level (high, medium, and low) are requested to calculate (1) BMI z-score, (2) energy expenditure, and (3) energy intake by the program. In this study, the children’s basic information is entered in advance by the investigators. A medium activity level is assumed for all children. BMI is calculated according to the standard formula: BMI=weight in kilogram/height in m^2^ and is subsequently transformed into the sex- and age-specific BMI z-scores according to German reference values [51]. Energy intake requirements are calculated based on the recommendations of the Research Institute of Child Nutrition, Dortmund, Germany, which, in turn, are based on the Food-Based Dietary Guidelines in Europe [52]. Individual energy expenditure is calculated according to the document *Human energy requirements*, a report of a joint Food and Agriculture Organization and World Health Organization and United Nations Organization expert consultation [53]. For children with a BMI z-score of less than −0.4, the program sets the energy requirements to the values calculated for children of the same age and sex and a BMI z-score of −0.4. Similarly, for children with a BMI z-score greater than 0.4, the program sets the energy requirements to the values calculated for children of the same age and sex and a BMI z-score of 0.4. Setting these cutoffs, we assure that the children do not pack portions that are connected with undereating and overeating. In the task, the children can choose from a large variety of foods from the food groups for breakfast, lunch, dinner, and two mid-meals. They can also choose to skip meals or mid-meals. The foods are presented as pictures for which the exact amount and energy contents and their assignment to the appropriate food group are entered in a database. Finally, individual visual and verbal feedback is given about the child’s energy balance and the distribution of the selected foods according to the food groups. The feedback is provided in a positive way. Besides the *food pyramid* recommendations [54,55], other state-of-the-art information retrieved from large epidemiological cohort studies, for example, the European Prospective Investigation into Cancer and Nutrition study [56] and from the World Cancer Research Fund International is included in this tool, for example, if a child exceeds the amount of food in the meat plus fish plus egg group, the recommendation will be to focus on fish and poultry and to avoid red meat.

##### Balloon Game

This game deals with the food pyramid and its food groups. At the beginning of the balloon game, the children are introduced to the food pyramid and nutrition circle [54,55]. To make the food groups clear, we invented animals representing the food groups: (1) fruit and vegetable group with a monkey with a banana, (2) grain food group with a hamster with an ear of corn, (3) dairy group with a cow with a milk bottle, (4) meat and fish group with a cat with a fish, (5) sweets and fats group with a bear with honey, and (6) liquids with a dolphin with a bottle of water. Next, a fun game starts where foods from the five food groups (without liquids) hanging from a balloon fly across the screen. On the bottom are five boxes labeled with the pictures of the food-group animals. The children then have to touch and pop the balloons to make the foods fall into the appropriate box. If the food falls into the wrong box, the food jumps out again and will appear with a balloon again. The game is over when all foods are in their correct food group box. In this module, we do not include the *dolphin group*. This last group is introduced in the module *Liquid rankings on the sugar scale*.

##### Foods Under the Microscope

In the next part of the game, the children learn about the main factor that induces satiety, which is the volume of food [57,58]. Next, the dietary energy density (DED) concept [59,60] is introduced, which organizes foods into 3 groups: (1) green foods with a DED up to 1.5 kcal/g, (2) yellow foods with a DED of 1.5 to 2.5 kcal/g, and (3) red foods with a DED above 2.5 kcal/g. According to the DED concept, green foods can be eaten in relatively large amounts and help to induce satiety, whereas red foods should be only eaten in small amounts and are mainly for enjoyment. Next, on the bottom of the screen, five box-like rectangles appear, labeled with the food-group animals. After clicking on one of these rectangles, five selected foods from the specific food group appear. The player can freely choose the foods to investigate them *under the microscope*. For every food, the proportion of water, fat, carbohydrates, protein, and fiber are displayed visually in test tubes in addition to the DED color information. The child also has the opportunity to obtain the information via additional audio texts. All foods must be investigated before the player is allowed to proceed. Next, a similar setup is used, but this time the child has to decide which food belongs to which DED color while the information about the test tubes is provided. In this game activity, the children collect points. They receive 3 points if the answer is correct the first time, 2 points if the answer is correct the second time, and 1 point if the answer is finally correct. After completion of every food group, a summary about the specific foods is given as audio text.

##### Liquid Rankings on the Sugar Scale

This game deals with the questions of (1) which liquids should be generally used for water intake (ie, water or sugar-free herbal and fruit teas), (2) how much liquid should be consumed daily (ie, as much as necessary to keep the urine transparent to very light yellow), (3) how much sugar is in liquids, (4) the impact of liquids on satiety [58,61]), and (5) how much exercise has to be done to burn the calories of 1 L coke or fruit juice. This game starts with the food pyramid concept, now introducing the dolphin group. Next, the game starts where a horizontal number line of lumps of sugar is provided on the top of the screen. On the left side of the screen, the following 1 L bottles of liquids are provided: water, sugar-free herbal tea, orange juice, orange lemonade, coke, apple spritzer, and ice tea. Each liquid appears in the middle of the screen when it is selected. Next, the player has to decide how many sugar lumps are *hidden* in the selected liquid. The player has three chances and receives verbal feedback about misestimation after every trial, for example, “there is much more sugar in this liquid,” “there is a little bit more sugar in the liquid,” “there is less sugar in the liquid,” and “the answer is correct.” After the third try (or before if the estimation was correct), the selected liquid is placed alongside its appropriate place on the number line of lumps of sugar. At the end of the game, the player has an overview of the sugar content in the liquids in relation to each other.

##### Kangaroo-Turtle Race

In this game, the player has to apply the knowledge about DED without having much time to think. A kangaroo (the computer) races against a turtle (the player). The kangaroo hops continuously, but the turtle is only fast if the rocket on its back is activated. In the middle of the screen, pairs of foods or liquids are presented in random order, with one food or liquid always having a higher DED than the other. The task is to choose the food or liquid with the lower DED. If the answer is correct, the rocket on the turtle is activated, and if the answer is false, a penalty of 3 seconds occurs. Some food or liquid pairs are already known from the previous games, and others are new to test the *transfer* of the DED concept to unknown foods. The winner is whoever passes the finishing line first. This is a fast game and is played three times in succession. Many food and liquid pairs are provided for this game so that during one game run, not all pairs will be displayed.

##### Bursting Bubble Game

This game provides information about eustress, distress, and coping strategies. The children then have the chance to burst bubbles on their tablet. The instruction is to breathe in slowly and breathe out slowly and loudly. The longer they breathe out, the larger the bubble will be. The children can burst the bubbles by tapping their finger on them. The basis for the functioning of the game is the microphone of the tablet. There should be no background noise in the room while the game is played.

##### Relaxation Story

This task begins with an introduction about eustress and distress and strategies to cope with stress. The idea of balancing activities in life is also introduced in this task. Next, the relaxation story starts. The children receive the instruction to sit conformably in a chair and to relax and simply listen. The story is about 2 min long.

##### Everyday Activities Task

The relaxation story is followed by a task where we measure the activities the children remember to do in their leisure time. The children are then motivated to reflect on their behavior. A set of typical activities is presented to the children (eg, playing soccer, riding a bike, and watching television). Next, they have the task to sort the activities they do into the categories “I do this every day,” “every week,” and “seldom.” The children also have the opportunity to add their own favorite activities. Finally, they are asked to plan their activities for the next week and to try to balance active and relaxing activities.

### Outcome Measures

Measurements were taken at baseline (T0), at 2 weeks after study commencement (T1), and at the 4-week follow-up (T2). The primary outcome of the study was the gain in knowledge (eg, nutrition and stress coping) measured by a self-constructed, pilot-pretested questionnaire tailored specifically for the serious game and to be analyzed between IG and CG at T0 versus T1. In a pilot study with 17 children, the game and the questionnaire were evaluated and further developed and adapted. It consists of 13 questions. The answers for the items were transformed to scales ranging from 0 to 100. Subsequently, sum scores were calculated and divided by the number of items resulting again in values between 0 and 100. The total knowledge test score includes all items; the DED score includes the items 3, 5, 7, and 9; the food pyramid score includes the items 1, 2, and 4, and the stress score includes the items 10, 11, 12, and 13. The questionnaire is provided in Multimedia Appendix 4.

Secondary outcomes were the maintenance of knowledge at T2, perception of knowledge gain, acceptance of the game, game module data dealing with the DED-P to support the results of the knowledge questionnaire, emotions during game play [62], game data regarding changes of dietary behavior [63,64], physical activity [65], and media consumption [66] measured by validated questionnaires. A detailed overview of the applied measurements for the secondary outcomes is provided below.

*Maintenance of knowledge* was measured by applying the knowledge questionnaire at the 4-week follow-up after T1. At this time, the CG had also just completed the intervention in view of ethical considerations. Maintenance of knowledge of IG was compared with the knowledge of CG, which had newly acquired the knowledge.

*Acceptance of the game (IG only at T1)* was measured by the three items “Overall, I like the game,” “Playing the game was fun,” and “Playing the game does not make me feel bored” answered on a 4-point scale, and the 2 items “Would you recommend the game to a friend?” and “Would you also like to play the game at home?” answered on a 3-point scale.

*Emotions during game play (IG only at T1)* was measured using the validated self-assessment manikin [62] as a nonverbal pictorial assessment (corresponding to a 5-point scale), which is applied to measure pleasure, arousal, and dominance associated with the children’s affective reaction during the game. The items were (1) “When I play the game I feel good (1)—bad (5),” (2) “When I play the game I feel relaxed (1)—aroused or excited (5),” and (3) “When I play the game I feel tall and strong (1)—small and weak (5).”

*Changes in dietary behavior* [63,64] *(IG and CG at T0 and T2)* were measured using the “Ernährungsmusterindex” (an index for healthy nutrition) [63]. This index consists of food items, which are considered to be indicators for healthy and unhealthy eating behaviors. The indicator food items used for this index are vegetables and fruits (cooked, raw, frozen, and tinned), whole-grain bread, soft drinks, fast food, chocolate, and snacks such as crisps or pretzel sticks. The questions were taken from the corresponding validated Food Frequency Questionnaire (FFQ) [64]. The children completed the questionnaire by themselves, with the parents also completing the same questionnaire for their children.

*Physical activity (IG and CG at T0 and T2)* was measured by a validated questionnaire [65], which was completed by the children themselves and by the parents on behalf of their children. The questionnaire consists of seven items, each with six answer options. Finally, a score was calculated, allowing categorizing the activity level into low, medium, and high.

*Media consumption (IG and CG at T0 and T2)* was measured by questions from the German Health Interview and Examination Survey for Children and Adolescents (KIGGS) questionnaire [66]. Four questions seek information on the average time the child spends watching television or playing video games or time spent on the computer during the week and at weekends. Each question has five response options.

Game data that assessed the application of the DED concept under time pressure (IG and CG, Kangaroo-Turtle race) were analyzed. The hit rate and reaction time for known foods were calculated using data from three consecutively conducted independent runs.

*Game data of the Kangaroo-Turtle race* (Section Game Modules and Multimedia Appendices 2-4), which tests the application of the DED-P under time pressure, were analyzed to support the results of the DED-P subscore from the knowledge questionnaire (IG and CG). The hit rate and reaction time for foods were calculated using data from three consecutively conducted independent runs.

#### Procedure of Measurements

Data were collected using standard operating procedures. All questionnaires were completed by all participating children simultaneously in a classroom setting with separated tables using paper and pencil. A teacher, trained school psychologist or medical student were present at all times during this process. No specific instructions were given to individual children. The parents completed the questionnaires at home using paper and pencil. The log data of the educational game were saved for additional analysis. The BMI z-score was calculated by calculating the BMI, on the basis of body weight measured by a calibrated scale and body height measured by a stadiometer and with reference to age- and sex-specific norms [51].

### Sample Size

On the basis of pilot data, the sample size was calculated regarding the primary outcome, that is, the group (IG vs CG)×time (T0 vs T1) interaction of knowledge. A univariate two-group repeated measures analysis of variance (ANOVA) will have 80% power to detect a variance among the group marginal means of 1.266, will have 99% power to detect a variance of 1.891 among the means of the 2 levels, and will have 99% power to detect an interaction between groups and levels with a variance of 0.766, assuming that the between-group error term is 3.92, the within-group error term is 1.77, the measure of *sphericity* of the covariance matrix, epsilon, is 1.00 when the significance level is 0.05, and the sample size in each of the two groups is 25. The sample size was adjusted for cluster randomization by assuming an interclass correlation coefficient of 0.035 and a mean of 15 pupils per cluster, resulting in a variance inflation factor of 1.49. This results in a sample size of 37 per study arm [67,68]. We assumed an additional dropout rate of 5% because of illness or other specifics at the school setting. Therefore, the final sample size for each group should be 39, resulting in 78 participants overall.

### Statistical Analysis

Data were analyzed using SPSS version 25 (IBM, Ehningen, Germany). Data are presented as means (standard deviation) and frequencies along with their percentages unless stated otherwise. Before test statistics, the normality distribution of data was tested using the Kolmogorov-Smirnov test, and the equality of variances between groups was tested using Levene test. Baseline differences between the groups were analyzed using unpaired *t* tests; if nonparametric, the differences were analyzed using the Mann-Whitney U test; or if nonmetric, the differences were analyzed using the chi-square test or the Fisher-Freeman-Halton test [69]. The Freeman-Halton test is an extension of Fisher exact test and was applied if the chi-square test was inappropriate because the frequencies in the cross-tables were less than 5 in a cell in greater than 20% of the cells.

The primary and secondary outcomes were calculated as unadjusted and adjusted estimates.

#### Unadjusted Model

The sample size was calculated as follows: the calculation of group (IG vs CG)×time (T0 vs T1 for knowledge and T0 vs T2 for other variables) interaction by 2×2 ANOVA. Different baseline levels for the DED score were controlled for using analysis of covariance (dependent variable: DED change score from T0 to T1, fixed factor: group, and covariable: DED score at T0). Nonmetric data were analyzed using the chi-square test or the Fisher-Freeman-Halton test (see the above specification) using the difference between the values at T0 and T2.

#### Adjusted Model Accounting for Cluster Randomization

To account for cluster randomization, an ANOVA with contrasts was performed using the school classes as factor. Planned contrasts were calculated not only for the IG versus CG school classes but also for a set of different class combinations to check the specificity and plausibility of observed contrast effects.

### Further Analysis

The data of the Kangaroo-Turtle race was analyzed across both groups (IG and CG) after game play using the Wilcoxon signed-rank test because of nonparametric data distribution.

#### Handling of Missing Data of Single Items in Questionnaires at Baseline (Secondary Outcomes)

At baseline, all parents and children filled in questionnaires as secondary outcome variables, but there were instances of single questions of questionnaires not being answered. The predictive mean matching (PMM) method [70-72] with five cases in each match set was used for missing data (mis-d) imputation of the FFQ, parent version (mis-d: 1.7%) and child version (mis-d: 0.9%); the activity questionnaires, parent version (mis-d: 0.9%) and child version (mis-d: 1.2%); and questions about media consumption (mis-d: 5.8%).

#### Intention-to-Treat Analysis and Handling of Missing Data of Single Items in Questionnaires at the End of Study Period

We analyzed the primary and secondary outcomes by intention-to-treat (ITT) analysis using the PMM with five cases in each match set. Mis-d of single items in the questionnaires was imputed as described for the handling of missing data at baseline. The percentages of mis-d of the questionnaires were as follows: FFQ parent version 10.4% and child version 3.7%, activity questionnaire parent version 8.9% and child version 3.8%, and questions about media consumption 12.5%. No mis-d imputation was performed for game acceptance where only descriptive data were reported.

## Results

### Participant Characteristics

**Figure 1:**
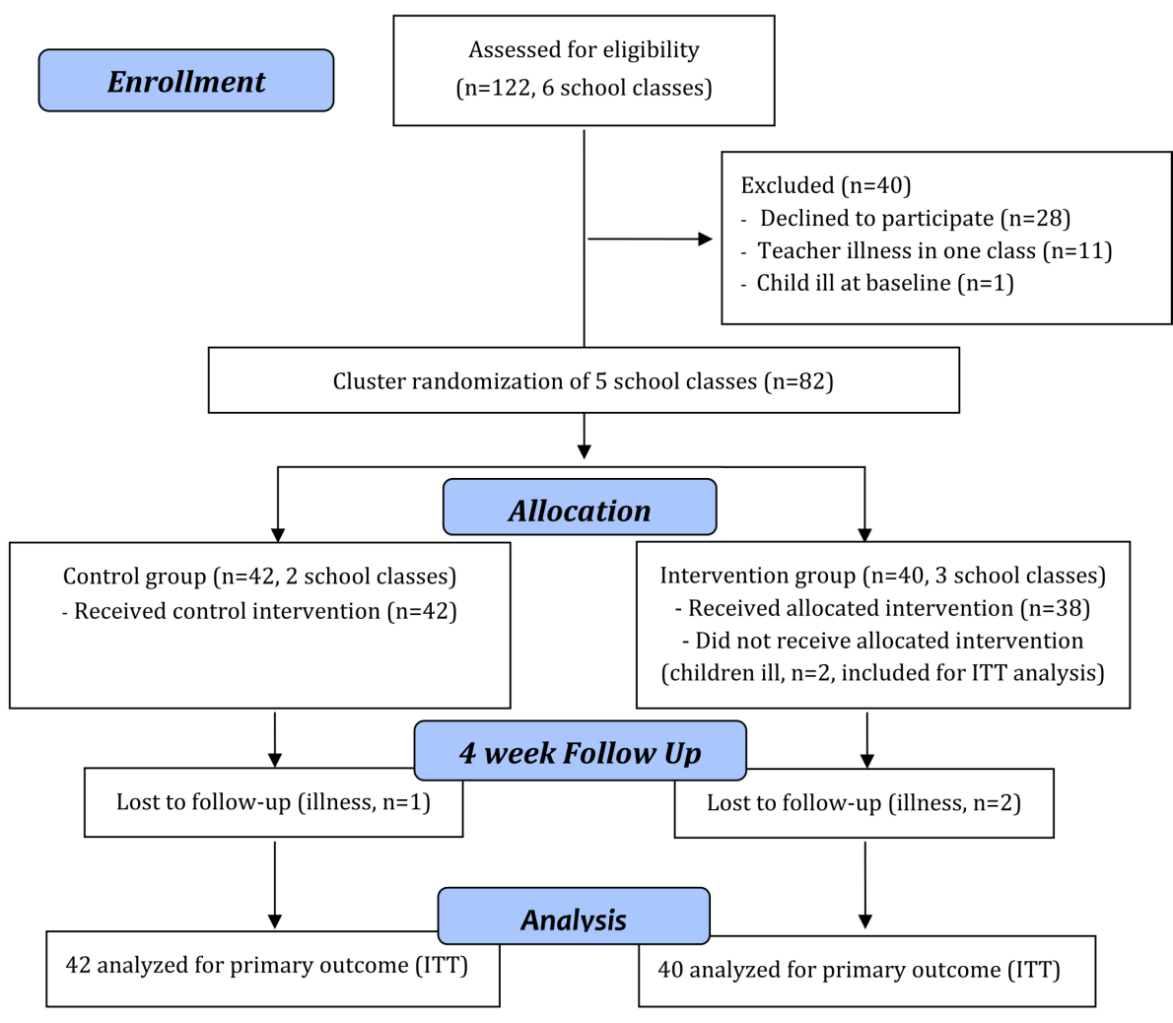
Consolidated Standards of Reporting Trials 2010 flow diagram for the Kids Obesity Prevention Study. ITT: intention-to-treat.

The flow of the participants in the study is shown in Figure 1. Of 122 children approached for the trial, 82 participated. After allocation to groups, 3 children dropped out because of illness (dropout rate: 3.6%). In Table 1, the baseline characteristics of the 82 children are shown, which were included in the ITT analysis. Overall, the study groups did not differ in sex, age, BMI z-score, and media consumption reported by parents, physical activity level and healthy eating index reported by children and parents, and the primary outcome knowledge about a healthy lifestyle with the focus on nutrition (total score). Regarding the knowledge subscores, the food pyramid score and the stress score did not differ between the groups. The knowledge subscore DED was higher in IG than in CG (U=587, *P*=.02).

### Outcomes

The data for the primary and secondary outcomes are presented in Tables 2 and 3. In addition, score changes from baseline are presented in Multimedia Appendix 5.

**Table 1:** Baseline characteristics of the study population.

Characteristics				Intervention group (n=40)		Control group (n=42)		*P* value
Age (years), mean (SD, range)				9.6 (4.9, 9-11)		9.7 (0.5, 9-11)		.38
**Sex (n)**								**.66**
	Male			19		24		
	Female			21		18		
Weight (kg), mean (SD, range)				37.3 (10.4, 24-65)		34.4 (9.2, 24-70)		.16
Height (cm), mean (SD, range)				143 (9, 129-166)		141 (6, 129-162)		.18
**BMI z-score, mean (SD, range)**				**0.21 (1.26, −2.44 to 2.65)**		**−0.02 (1.08, −2.07 to 2.57)**		**.34**
	Normal weight, n (%)			27 (68)		35 (83)		
	Thinness, n (%)			2 (5)		1 (2)		
	Overweight, n (%)			7 (17)		4 (10)		
	Obesity, n (%)			4 (10)		2 (5)		
**Language spoken at home (n)**								**.80**
	German			17		23		
	German and foreign language			16		14		
	Foreign language			7		5		
**Knowledge score, mean (SD)**								
	Percentage of total score (primary outcome)			53 (10)		50 (11)		.13
	Percentage of food pyramid score			50 (13)		49 (14)		.70
	Percentage of dietary energy density score			51 (18)		41 (19)		.02
	Percentage of stress score			56 (17)		59 (20)		.52
**Healthy Nutrition Index**								
	**Reported by parents**							
		Score, mean (SD)	9.2 (2.0)		9.8 (2.1)		.91	
		Unfavorable, n (%)	1 (3)		2 (5)			
		Neutral, n (%)	29 (72)		23 (55)			
		Favorable, n (%)	10 (25)		17 (40)			
	**Reported by children**							
		Score, mean (SD)	8.8 (2.1)		8.9 (2.2)		.13	
		Unfavorable, n (%)	1 (3)		4 (10)			
		Neutral, n (%)	29 (72)		30 (71)			
		Favorable, n (%)	10 (25)		8 (19)			
**Physical activity level**								
	**Reported by parents**							
		Score, mean (SD)	−0.9 (2.8)		−0.9 (3.2)		.97	
		Low, n (%)	15 (38)		17 (40)			
		Medium, n (%)	19 (47)		17 (41)			
		High, n (%)	6 (15)		8 (19)			
	**Reported by children**							
		Score, mean (SD)	−1.6 (2.7)		−1.5 (3.2)		.86	
		Low, n (%)	17 (42)		18 (43)			
		Medium, n (%)	20 (50)		19 (45)			
		High, n (%)	3 (8)		5 (12)			
**Media consumption**								
	**Watching TV or video films per day reported by parents, n (%)**						**.63**	
		≤0.5 hours	13 (33)		19 (45)			
		1-2 hours	26 (65)		22 (53)			
		≥3 hours	1 (2)		1 (2)			
	**Doing gaming activities at a computer per day reported by parents, n (%)**						**>.99**	
		≤0.5 hours	37 (92)		38 (90)			
		1-2 hours	3 (8)		4 (10)			
		≥3 hours	0 (0)		0 (0)			

**Table 2:** Outcomes from the intention-to-treat analysis.

Outcomes^a^			IG^b^ (n=40)	CG^c^ (n=42)	IG versus CG	Contrast IG versus CG *P* value		Effect size Eta^2^/Cramers V^d^	
**Knowledge, mean (SD); 95% CI**									
	Percentage of total score (primary outcome)		69 (11); 65 to 72	52 (12); 49 to 56	17 (12 to 22)	<.001		0.235	
	Percentage of food pyramid score		77 (12); 74 to 81	54 (12); 50 to 57	23 (18 to 28)	<.001		0.337	
	Percentage dietary energy density score		64 (17); 58 to 69	46 (22); 40 to 53	18 (1 to 27)	.097		0.131	
	Percentage of stress score		68 (18); 63 to 74	58 (24); 51 to 66	10 (1 to 19)	<.01		0.086	
**Physical activity level**									
	**Reported by parents**								
		Score, mean (SD); 95% CI	−2.1 (2.8); 3.0 to −1.2	−2.7 (2.7); −3.6 to −1.9	−0.6 (−0.6 to 1.8)	.48		0.012	
		Low, n (%)	21 (53)	27 (64)	−6 (−11)				
		Medium, n (%)	17 (42)	12 (29)	5 (13)				
		High, n (%)	2 (5)	3 (7)	−1 (−2)				
	**Reported by children**								
		Score, mean (SD); 95% CI	−2.7 (3.1); −3.6 to −1.7	−2.6 (3.4); −3.7 to −1.6	0.1 (−1.3 to 1.5)	.87		0.000	
		Low, n (%)	25 (62)	25 (60)	0 (0)				
		Medium, n (%)	13 (33)	12 (28)	1 (5)				
		High, n (%)	2 (5)	5(12)	−3 (−7)				
**Healthy Nutrition Index**									
	**Reported by parents**								
		Score, mean (SD); 95% CI	10.0 (2.7); 9.1 to 10.8	10.2 (2.0); 9.5 to 10.7	0.2 (−0.8 to 1.2)	.31		0.014	
		Unfavorable, n (%)	2 (5)	0 (0)	2 (5)				
		Neutral, n (%)	20 (50)	26 (62)	−6 (−12)				
		Favorable, n (%)	18 (45)	16 (38)	−2 (−7)				
	**Reported by children**								
		Score, mean (SD); 95% CI	9.5 (2.2); 8.8 to 10.1	9.3 (2.5); 8.5 to 10.1	0.2 (−0.9 to 1.2)	.68		0.002	
		Unfavorable, n (%)	0 (0)	3 (7)	−3 (−7)				
		Neutral, n (%)	25 (62)	27 (64)	−2 (−2)				
		Favorable, n (%)	15 (38)	12 (29)	3 (9)				
**Media consumption**									
	**Watching TV or video films per day reported by parents, n (%)**								**0.045^d^**
		<0.5 hours	14 (35)	21 (50)	−7 (−15)	N/A^e^			
		1-2 hours	24 (60)	16 (38)	8 (22)	N/A			
		≥3 hours	2 (5)	5 (12)	−3 (−7)	N/A			
	**Doing gaming activities at a computer per day reported by parents, n (%)**								**0.141^d^**
		≤0.5 hours	37 (93)	34 (81)	3 (12)	N/A			
		1-2 hours	2 (5)	7 (17)	−5 (−12)	N/A			
		≥3 hours	1 (2)	1 2)	0 (0)	N/A			

^a^For all outcomes, except for media consumption, the data are presented as mean (standard deviation) and 95% CIs for the IG and CG and for the changes between the groups (IG vs CG). For the physical activity level, the Healthy Nutrition Index, and media consumption, the data are also presented as sample size and percentage of the corresponding category. The *P* values of the contrasts IG versus CG of the ANOVA of the adjusted model along with the effect sizes are presented.

^b^IG: intervention group.

^c^CG: control group.

^d^The effect size according to Cramers V.

^e^Not applicable.

**Table 3:** Complete overview of statistics from the intention-to-treat analysis.

Outcome^a^				Unadjusted group effect, *P* value		Overall class effect, *P* value		Contrast IG^b^ versus CG^c^ (95% CI of contrast)		Contrast IG versus CG, *P* value		Effect size (Eta^2^/Cramers V^d^)
**Knowledge**												
	Percentage of total score (primary outcome)			<.001		<.001		0.39 (0.238 to 0.542)		<.001		0.235
	Percentage of food pyramid score			<.001		<.001		0.686 (0.494 to 0.878)		<.001		0.337
	Percentage of dietary energy density score			.001		.19		0.241 (−0.045-0.527)		.097		0.131
	Percentage of stress score			.008		.05		0.379 (0.109 to 0.648)		<.01		0.086
**Physical activity level**												
	**Reported by parents**											
		Score	.320		.48		1.270		.48		0.012	
		Categories	.951				(−2.325 to 4.866)				0.048^d^	
	**Reported by children**											
		Score, n	.883		.96		0.278		.87		0.000	
		Categories	.843				−3.121 to 3.678				0.079^d^	
**Healthy Nutrition Index**												
	**Reported by parents**											
		Score	.297		.06		−1.453		.31		0.014	
		Categories	.353				(−4.304 to 1.398)				0.163^d^	
	**Reported by children**											
		Score	.681		.58		0.673		.68		0.002	
		Categories	.819				(−2.537 to 3.884)				0.072^d^	
**Media consumption**												
	Watching TV or video films per day in hours reported by parents			.948 (categories)		N/A^e^		N/A		N/A		0.045^d^
	Doing gaming activities at a computer per day in hours reported by parents			.676 (categories)		N/A		N/A		N/A		0.141^d^

^a^The complete statistics for the outcomes of the study are presented for the unadjusted and adjusted models, the latter being an analysis of variance with contrasts.

^b^IG: intervention group.

^c^CG: control group.

^d^Cramers V effect size.

^e^Not applicable.

#### Primary Outcome: Knowledge of the Game

The primary outcome was the gain in knowledge (total knowledge test score). Knowledge increased significantly from T0 to T1 when comparing IG with CG in the unadjusted and adjusted model. All subscores increased significantly in the unadjusted model from T0 to T1. In the adjusted model, the subscores *food pyramid* and *stress score* increased, and a clear trend was shown for the DED score from T0 to T1. In addition, all scores improved slightly from T0 to T1 regardless of the group allocation (total knowledge test score: *F*_1,80_=49.597, *P*>.001; food pyramid score: *F*_1,80_=98.600, *P*<.001; DED score: *F*_1,79_=11.921, *P*=.001; and stress score: *F*_1,80_=6.891, *P*=.01).

#### Secondary Outcomes

*Maintenance of knowledge* was tested at the 4-week follow-up from T1. The gain in knowledge was maintained in IG over a 4-week period (from T1 to T2) and was comparable with the knowledge of CG, which had just completed the intervention (see the subsection Outcome Measures under the Methods section), and no differences between the groups were evident (total knowledge test score for IG: 69.7% [SD 11.0] vs CG: 68.3% [SD 18.7]; food pyramid score: IG 78.9% [SD 7.9] vs CG: 77.4 [SD 11.2]; DED score: IG: 68.5% [SD 14.1] vs CG: 65.2 [SD 16.0]; and stress score: IG: 69.3 [SD 22.2] vs CG: 68.3 [SD 18.7

#### The Dietary Energy Density Principle Under Time Pressure (Intervention Group and Control Group After Game Play)

To support the data of the DED score, we analyzed the game data of both groups where the DED-P had to be applied under time pressure (Kangaroo-Turtle race) after DED education. Under these conditions, the children were able to classify unknown foods equally to known foods according to their DED (hit rate: 72.5% [SD 14.9] vs 70.5% [SD 14.2]; T=−1127; *P*=.260). However, it took the children longer to make a decision when unknown foods were presented in comparison with known foods (1542 ms [SD 346] vs 1495 ms [SD 465]; T=−3484; *P*<.001).

#### Acceptance of the Game and Feelings During Game Play (Intervention Group Only)

Overall, 92% (37/40) of participants in IG responded that they liked the game very much or much, 3% (1/40) found it okay and 5% (2/40) were mis-d. Furthermore, 90% (36/40) of participants in IG agreed that playing the game was fun, whereas 5% (2/40) agreed partly and 3% (1/40) disagreed and 3% (1/40) were mis-d. In addition, 85% (34/40) of participants in IG agreed that it did not make them feel bored, whereas 10% (4/40) agreed partly and 5% (2/40) disagreed or were mis-d. Furthermore, 80% (32/40) of participants in IG also would like to play the game at home, 7.5% were not sure (n=3), and 13% (5/40) would not or were mis-d. More than two third of participants in IG (31/40, 78%) would recommend the game to a friend, 15% (6/40) were not sure, and 8% (3/40) would not or were mis-d. Using a nonverbal pictorial assessment, the children reported that overall they felt good, strong, and relaxed when playing the game. For question 1, the mean was 1.1 (SD 0.3); for question 2, it was 1.8 (SD 1.2); and for question 3, it was 1.8 (SD 1.0).

#### Dietary Behavior

Dietary behavior was assessed using a Healthy Nutrition Index (HNI). At baseline, HNI was predominantly neutral or favorable as reported by the children and their parents. From T0 to T2, no significant changes were observed between the groups neither in HNI reported by the children themselves nor by their parents (Tables 2 and 3 and Multimedia Appendix 5). However, regardless of the group allocation, HNI improved from T0 to T2 as reported by parents (*F*_1,80_=4.916; *P*=.029) and children (*F*_1,80_=10.863; *P* trend=.057).

#### Physical Activity

At baseline, most of the children had a low or moderate physical activity level. No changes were observed in the time course between the different group allocations as reported by children and their parents (Tables 2 and 3 and Multimedia Appendix 5). However, from T0 to T2, the physical activity level decreased across all groups according to children (*F*_1,80_=15.924; *P*<.001) and parents (*F*_1,80_=22.153; *P*<.001).

#### Media Consumption

The duration of watching TV or video films on weekdays was between 0 and 30 min or 1 to 2 hours for most children at baseline. Gaming activities duration at a computer on weekdays was between 0 and 30 min for most children at baseline. No significant changes were found between the groups in the time course. However, changes in media consumption independent of group allocation were found for both investigated items (each *P*<.001, Fisher exact test, has no test value). The most prominent changes were found for watching TV or video films, where almost balanced bidirectional changes in consumption occurred in 35% to 40% of children (Tables 2 and 3 and Multimedia Appendix 5).

## Discussion

### Principal Findings

#### Knowledge

To our knowledge, KOP is the first health game that addresses three core areas of obesity prevention and treatment: nutrition, physical activity, and stress coping. In the nutrition section, it is the first that extensively focuses on the DED concept.

The main hypothesis was verified, showing that children gained sustainable knowledge about the food pyramid concept, the DED concept (including the topic of liquids), and about stress and stress-coping strategies after game play. Second, we hypothesized that after the 4-week follow-up, the knowledge scores would be higher than at baseline but lower in comparison with the scores directly after the game. Interestingly, 4 weeks after the intervention, the knowledge level was similar to the level directly after intervention. This was unexpected considering that knowledge is quickly lost if not consciously reviewed from time to time [73,74]. This is a promising result, which may be explained by the high level of challenging interactions, repetitions, and self-reflective tools applied in the game. It also shows how powerful games can be for standardized knowledge transfer when correctly designed and when topics are presented appealingly to children (and adolescents and adults) [75]. It is possible that the knowledge transfer was only sustainable because the children might have been more receptive to the topic and solidified the knowledge in thought and practice after the intervention. This was not measurable at the HNI level, however, as this did not differ between the groups but improved in the course of time in both groups, as will be discussed below. The maintenance of knowledge has not been tested by other serious games targeting nutrition and obesity [29], except in a recent report in which a 2-week follow-up nutrition knowledge test was performed where at the follow-up, similar results were observed for IG and CG. This was explained by (1) high baseline knowledge levels of both groups not leaving much room for knowledge gain and (2) CG having an extremely good learning curve by repeated exposure to the same tests [47]. In concordance, we also saw a time-dependent knowledge effect independent of the group, although this was marginal.

In contrast to the abovementioned study, in this study, the knowledge testing after baseline was after 4 weeks, and the knowledge questionnaire was designed using a wide range from simple to difficult questions, thereby leaving room for grading of knowledge and making it difficult to score highly if certain content was not explicitly taught.

#### Understanding and Applying the Dietary Energy Density Principle

The third hypothesis stated that the children could understand and apply the DED-P after game play. At knowledge level, the subscore DED did not reach significance between the groups in the adjusted model, but a clear trend was observed (*P*<.1). This observation may be because of (1) that the questionnaire was designed for the total score and not explicitly for the subscores (eg, fewer points can be collected with this score when compared with the food pyramid score) and (2) that the IG already scored significantly higher at baseline regarding the knowledge of DED-P. More importantly, the children were able to apply their DED knowledge by transferring it to unknown foods when tested under time pressure, as 70% were able to correctly categorize the food pairs according to their DED. No reference values from the literature could be retrieved, either for children or adults. Hermmans et al [47] included a game on healthy and unhealthy food pairs (without defining what healthy and unhealthy are) where some pairs may have indirectly presented pairs of different DEDs. Here, the hit rate for *healthy food choices* was around 75%. The observed 70% correct categorization rate for food pairs under time pressure appears substantial. The finding that the correct categorization rate was similar between known and unknown food pairs is also promising. Although it took slightly longer to make the decision when unknown food pairs were presented, the principle was shown to have been understood, and a transfer of that knowledge to other foods was possible. This may be important when making subconscious decisions regarding food selection [76]. We have no baseline data for this DED-P game module (Kangaroo-Turtle race) because the learning effect of the module itself is assumed to be high and, therefore, could not have been applied in CG or IG before intervention. 

#### Game Acceptance and Behavior

Hypothesis four was verified by showing that children felt good during game play and game acceptance was high. Finally, we tested behavioral aspects, although we hypothesized no changes at the behavioral level would occur because of the study focus and design. Behavioral measures were assessed at baseline and T2. In the course of the study period, we observed bidirectional changes of media consumption in both groups but no differences between the groups. We observed behavioral changes at the physical and dietary levels across both groups, reported independently by the children and their parents. Physical activity decreased in roughly 40% or 30% of children depending on the reporting source, whereas in 10%, it increased. Thus, 20% to 30% of children decreased their activity level, which may be explained by the fact that the study commenced in autumn after 6 weeks of summer holidays. The decreasing physical activity levels observed may be related to more inactivity during school time, shorter daylight periods, and a temperature drop. The intervention itself was not associated with a change in physical activity. This is in keeping with the short study period and the game design being unlikely to effect a change in physical activity level. The motion control was used to avoid long times of inactivity during game play but did not focus on increasing physical activity as classical exergames do [29].

As mentioned previously, HNI improved slightly over the course of the study. Approximately 20% of the children improved in category, whereas around 10% showed a deterioration in category. It must be noted that the baseline HNI in about 95% of children was already either neutral or favorable, and category changes appear to be rather moderate because the proportion of children in the unfavorable category did not increase. The changes observed over time may be spontaneous because of real or perceived dietary changes occurring in the context of the switch between holidays and school routine together with seasonal factors, may reflect participation in this health study, or may involve a combination of these factors. CG also received material about a healthy lifestyle, which may have motivated some families to maintain a healthier diet. The HNI data here are in line with the findings of a national health survey (KIGGS), which used the same instrument for dietary assessment [63].

Overall, it is a positive finding that taking part in this health game study improved dietary behavior measured with HNI in families, but for sustainable lifestyle changes in children, it is important to promote child-parent interactions. Parents are role models for their children and have the power to influence their behavior and thinking or help to solidify new patterns of activity in children and adolescents [77,78].

### Limitations and Outlook

To date, there is a lack of health game interventions, which include parents [29].

This is a limitation of this study, as we only involved parents for data collection. Second, our follow-up period was short, and we did not include behavioral tests, for example, for measuring eating behavior. We also have to consider that knowledge improvements about health issues do not necessarily result in changes at the behavioral level. Owing to the nature of this intervention, no double-blind and placebo-controlled study design was possible to evaluate the game, and this may have influenced the results.

To address some of these limitations, we are in the process of undertaking a further randomized controlled trial to evaluate the game regarding (1) its acceptance and efficacy in parents and their children and (2) whether the children can benefit by the involvement of their parents. A 6-month follow-up is also included in this study. We also plan to conduct trials where the efficacy and acceptance of the game are tested in established inpatient and outpatient treatment settings for children. By involving not only the children but also their parents, these studies aim to improve parental support for their children and compliance of the children. Making the topics of *nutrition* and *stress and stress regulation* more appealing using modern media should improve the outcome of the children in these programs. Finally, the approach of supporting individual lifestyle changes should be complemented by community-based and environment-oriented approaches to combat obesity [79-81].

### Conclusions

Taken together, KOP is the first serious game for children addressing the three topics of nutrition, physical activity, and stress coping. To our knowledge, it is also the first game, which extensively targets the DED concept in the area of nutrition, in addition to playfully dealing with stress coping. The game was highly accepted by children, sustainably increased their knowledge of the topics addressed, and could be a useful tool for further studies and education.

## Appendix

Multimedia Appendix 1 Overview of topics and modules.

Multimedia Appendix 2 Screeenshots of the game.

Multimedia Appendix 3 Video of the game.

Multimedia Appendix 4 Questionnaire.

Multimedia Appendix 5 Results-changes from baseline.

Multimedia Appendix 6 CONSORT-eHEALTH checklist (V 1.6.1).

## Abbreviations

ANOVA: analysis of variance
CG: control group
DED-P: dietary energy density principle
FFQ: Food Frequency Questionnaire
HNI: Healthy Nutrition Index
IG: intervention group
ITT: intention-to-treat
KIGGS: German Health Interview and Examination Survey for Children and Adolescents
KOP: Kids Obesity Prevention
mis-d: missing data
PMM: predictive mean matching
