# Supplementary material for: The Kids Obesity Prevention Program: Cluster Randomized Controlled Trial to Evaluate a Serious Game for the Prevention and Treatment of Childhood Obesity
Source: J Med Internet Res. 2020 Apr 24;22(4):e15725. doi: 10.2196/15725 (PMC7210499; doi:10.2196/15725)
Supplement: Multimedia Appendix 1 [file jmir_v22i4e15725_app1.pdf]

Supplement: Overview of the addressed areas, topics and modules of the game

Core areas	Nutrition	Physical Activity	Stress and Stress Coping
**Topics**	Food groups/food pyramide<br>Energy density concept<br>Sugar in liquids<br>Self-reflexion of daily food intake	Moderate physical activity	Eustress and distress<br>Coping with stress<br>Reflexion and planning of everyday activities
**Game Modules**	Baloon game[1]<br>Pack your backpack with food[1,2]<br>Foods under the microscope[1]<br>Liquid rankings on the sugar scale[2]<br>Kangaroo-Turtle race[2]	Motion control of the game[1,2]	Bursting bubble game[1]<br>Relaxation story[2]<br>Everyday activities task[2]

1=Modules of game session 1
2=Modules of game session 2
