# Supplementary material for: The Kids Obesity Prevention Program: Cluster Randomized Controlled Trial to Evaluate a Serious Game for the Prevention and Treatment of Childhood Obesity
Source: J Med Internet Res. 2020 Apr 24;22(4):e15725. doi: 10.2196/15725 (PMC7210499; doi:10.2196/15725)

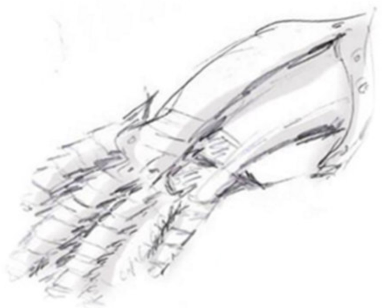

KOP – Introduction to story

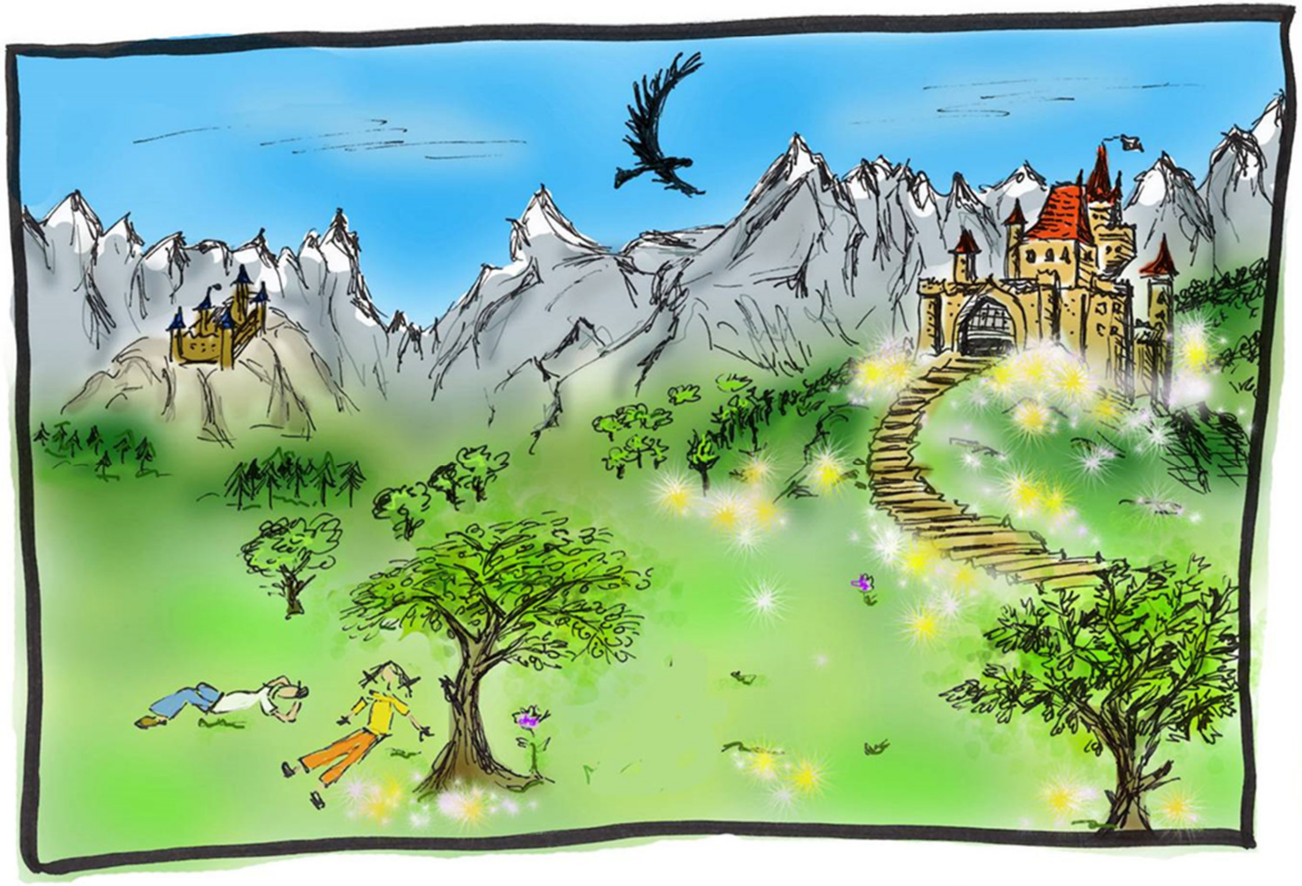

Navigation through the world

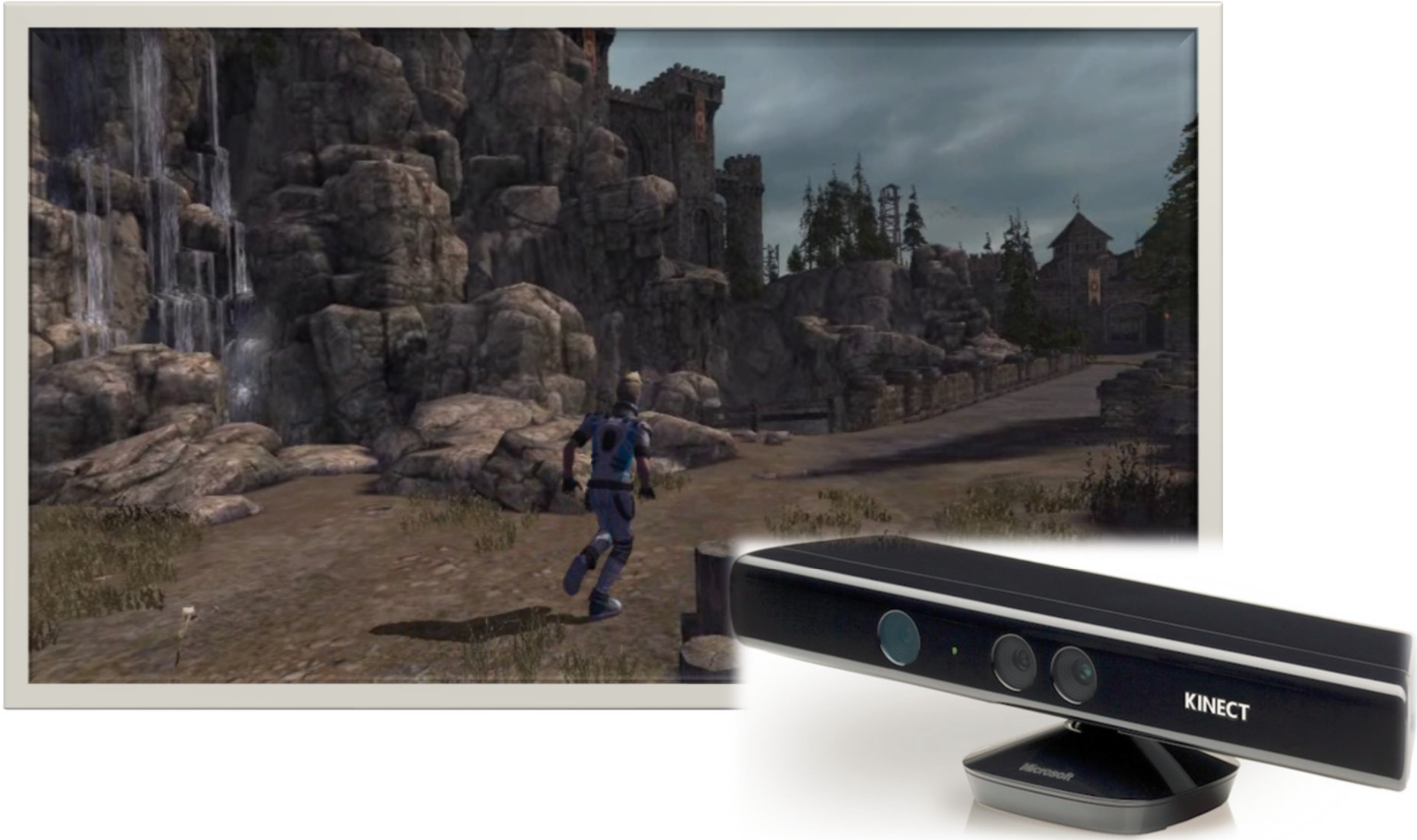

Game module – Pack your backpack with food

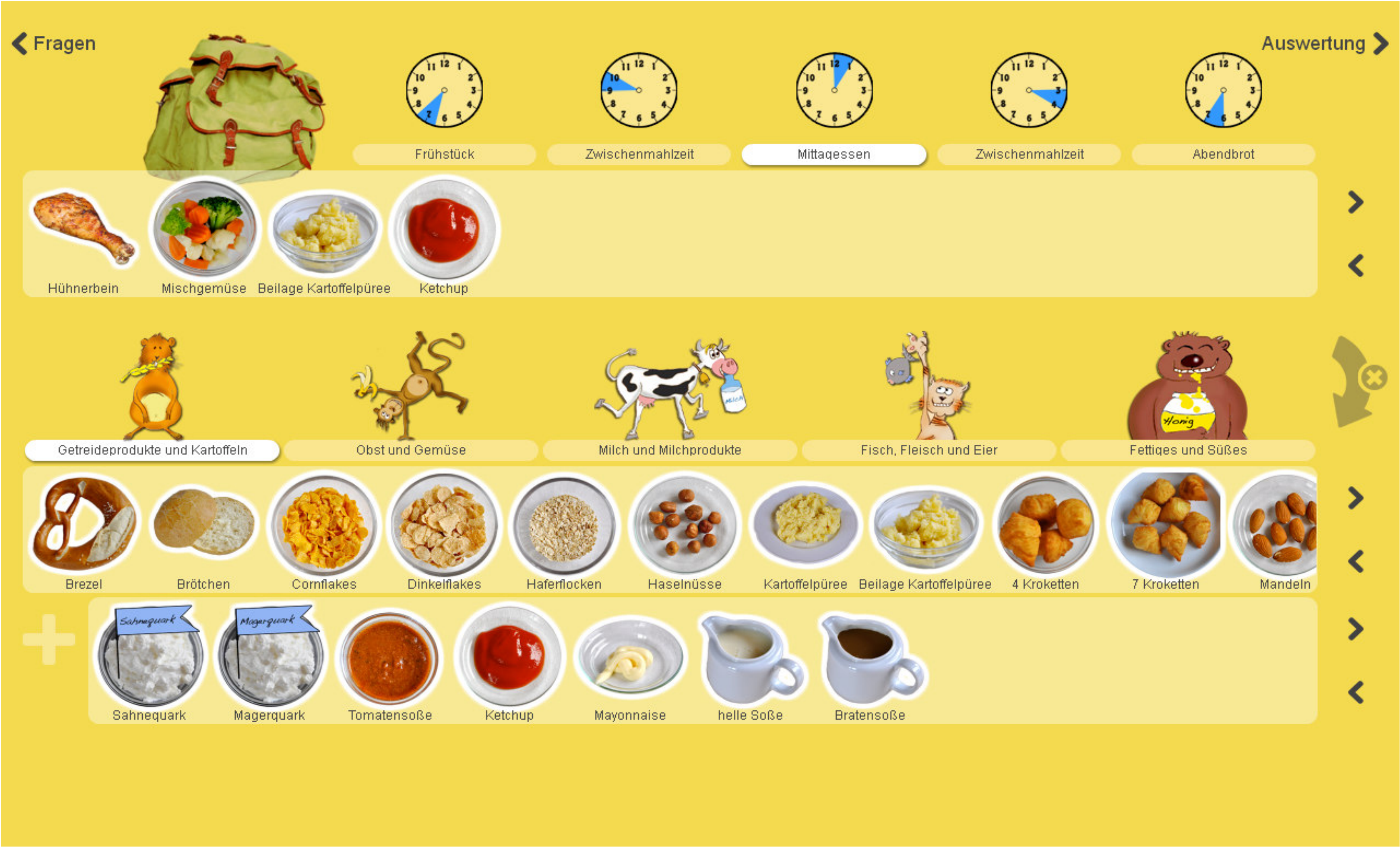

Game module – Pack your backpack with food

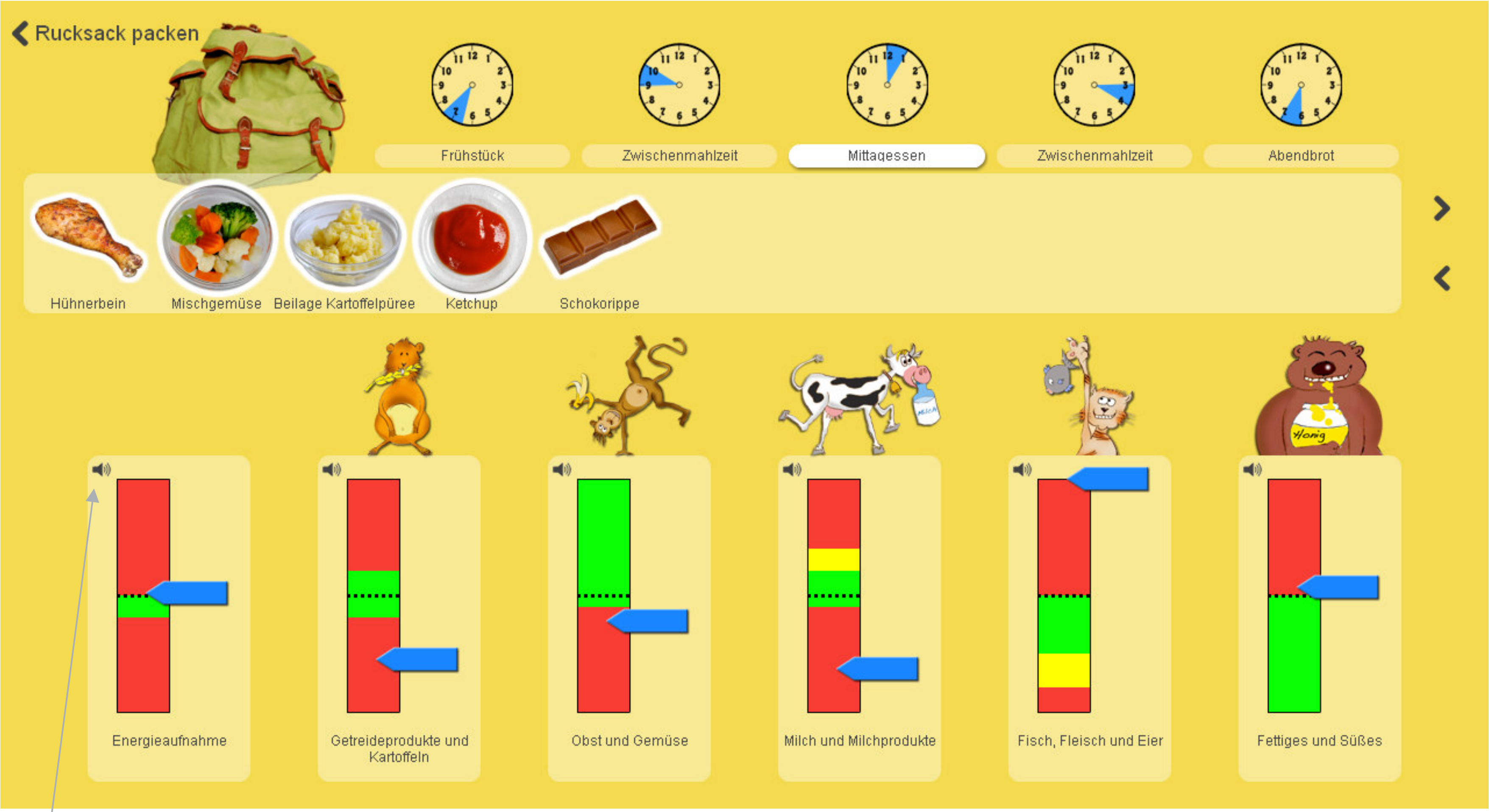

Audio for detailed analysis and proposing alternatives

Game module – Balloon game

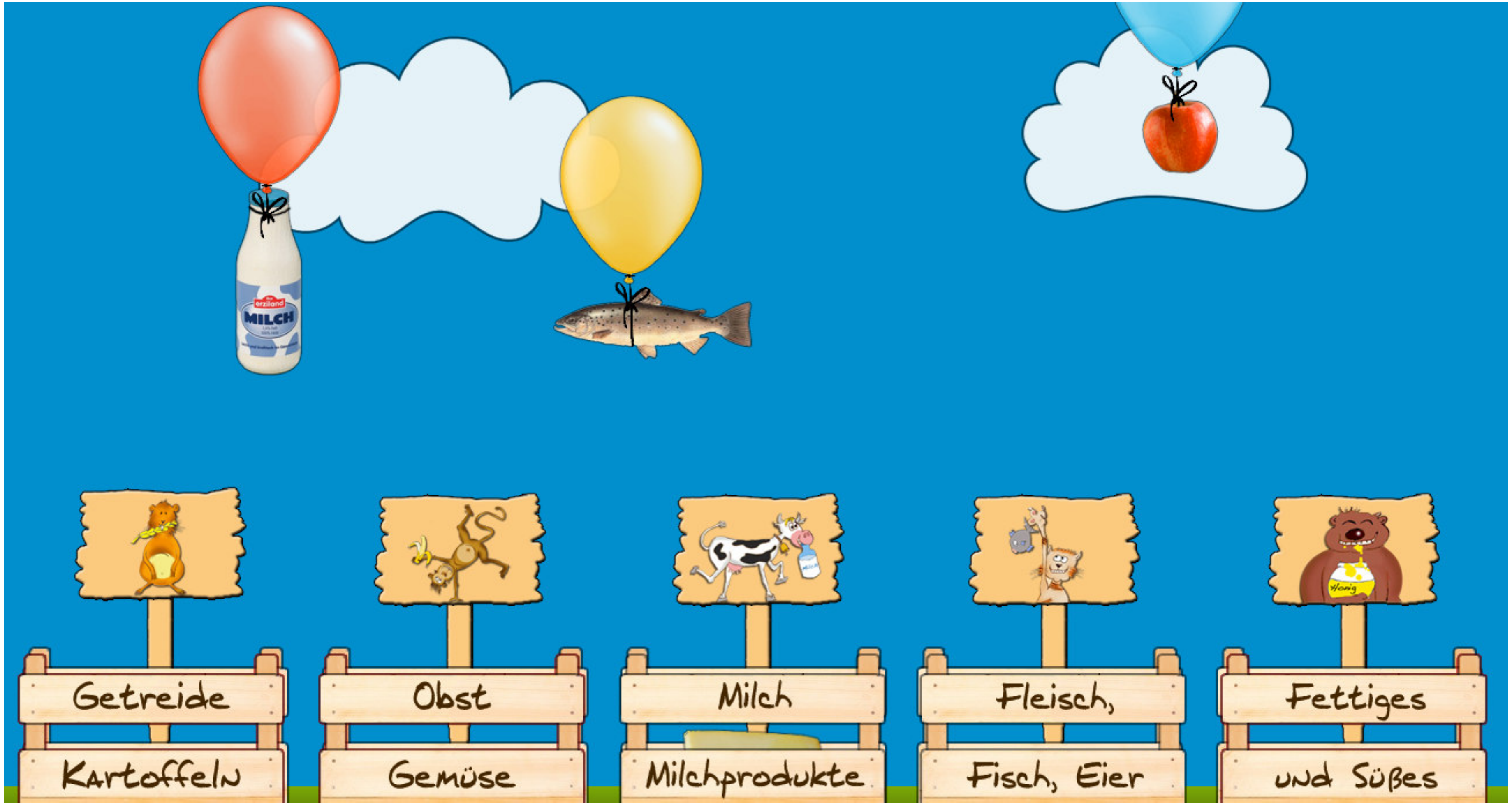

Game module – Foods under the microscope: What causes satiety?

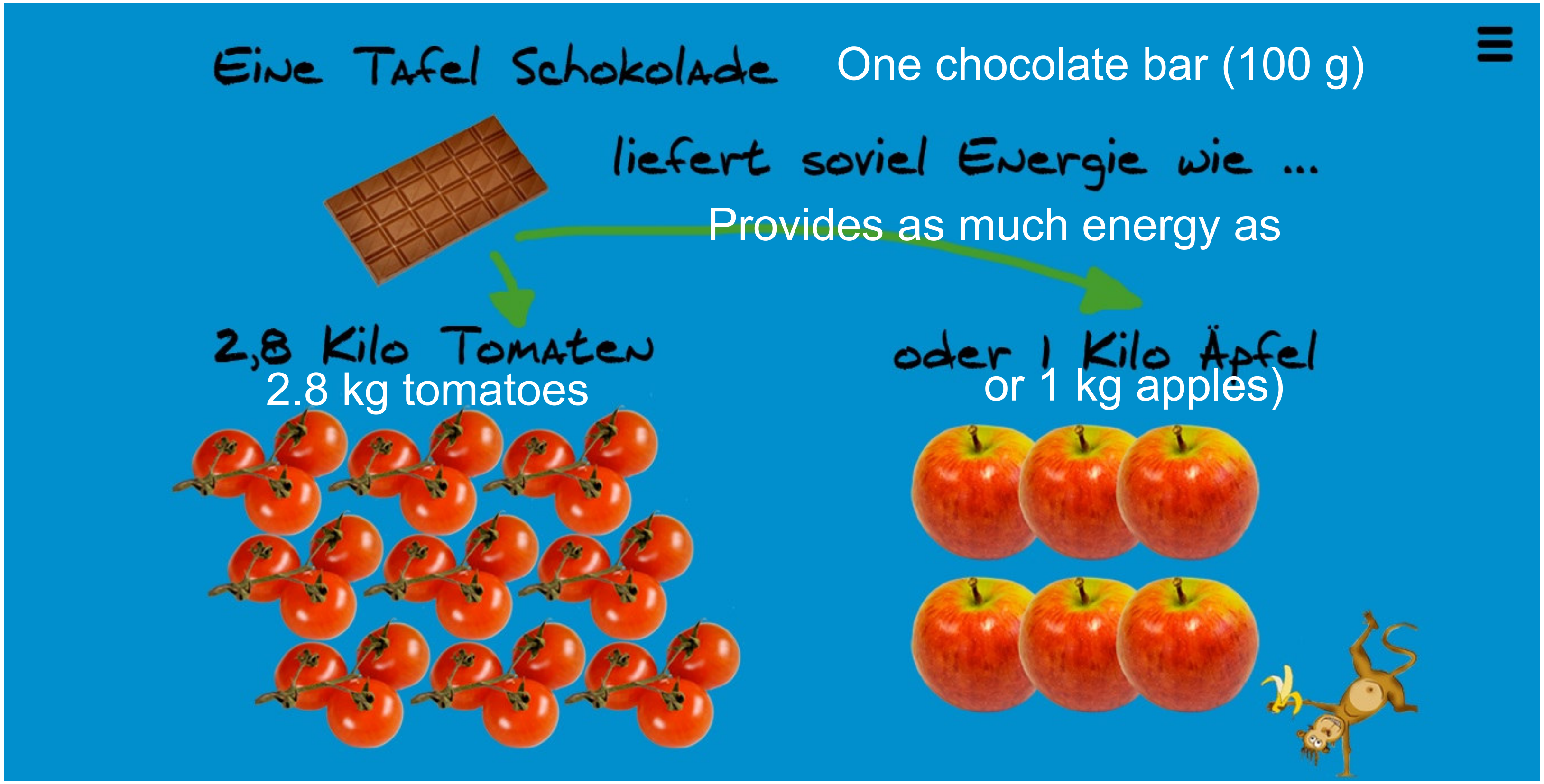

So it is not the energy in foods which causes satiety, it is in the first place the volume of foods.

Foods under the microscope (first part where you learn about the foods): green foods – eat plenty

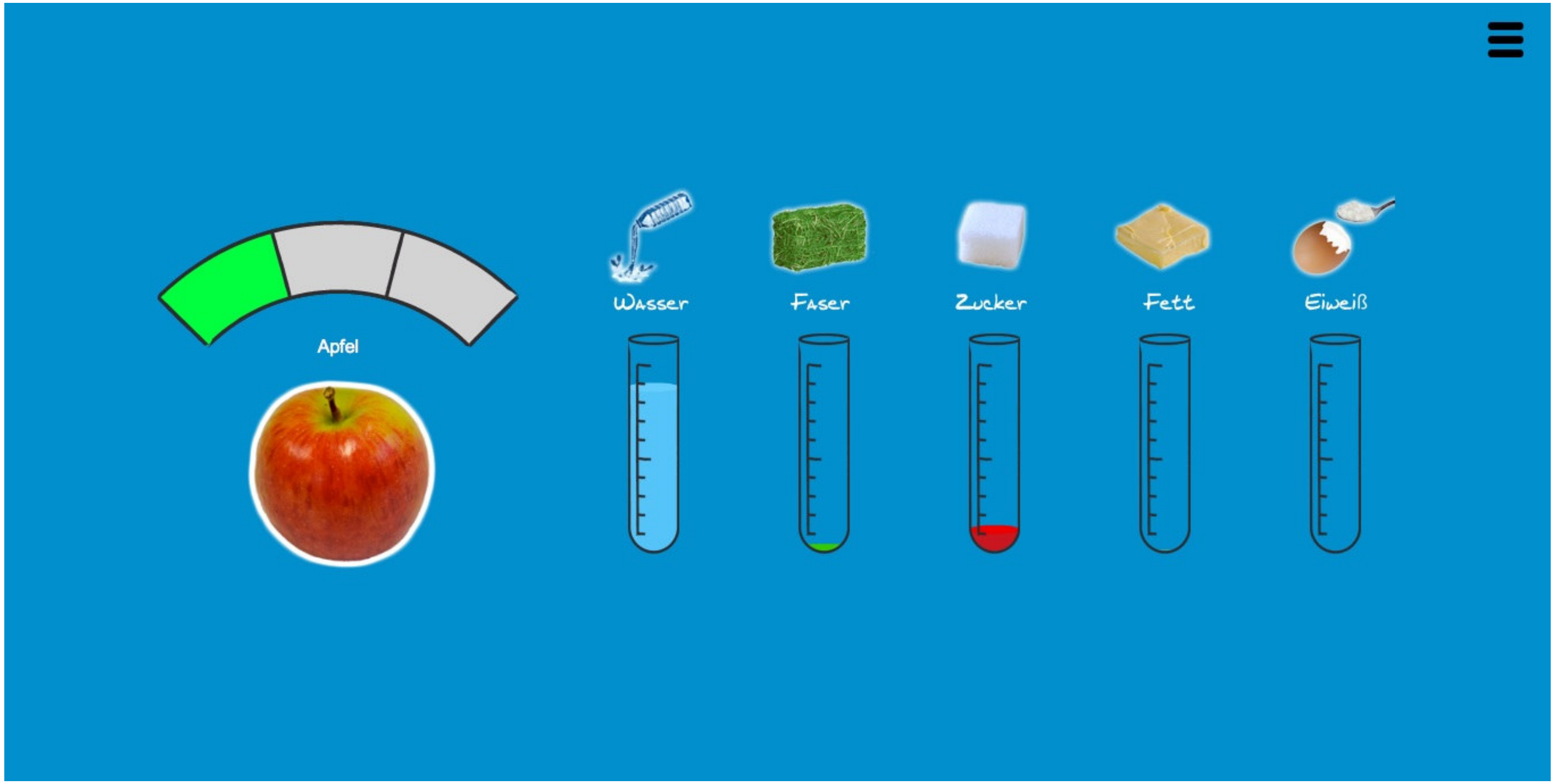

Foods under the microscope (first part where you learn about the foods): red foods – eat small quantities (for pleasure)

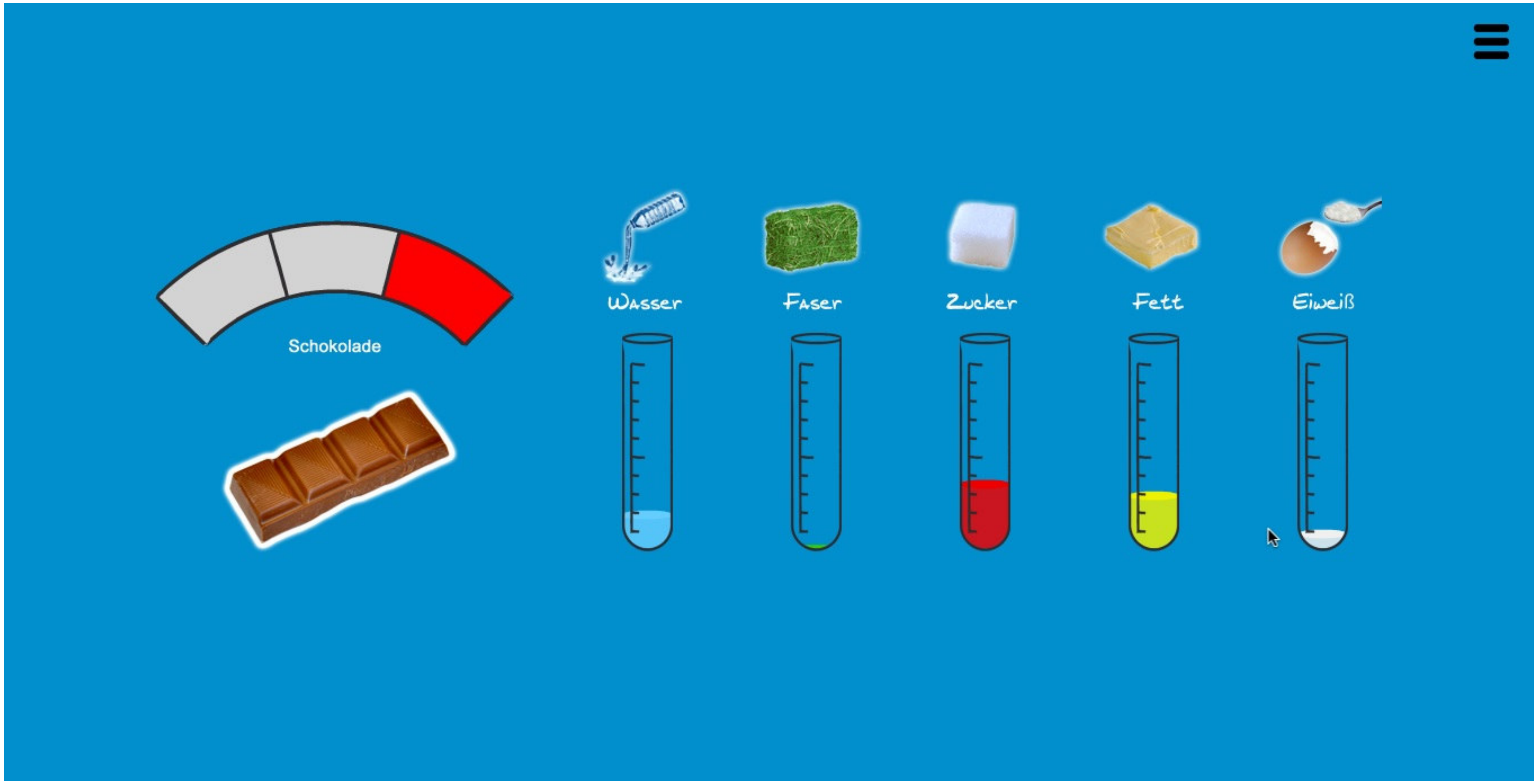

Foods under the microscope (second part): Game

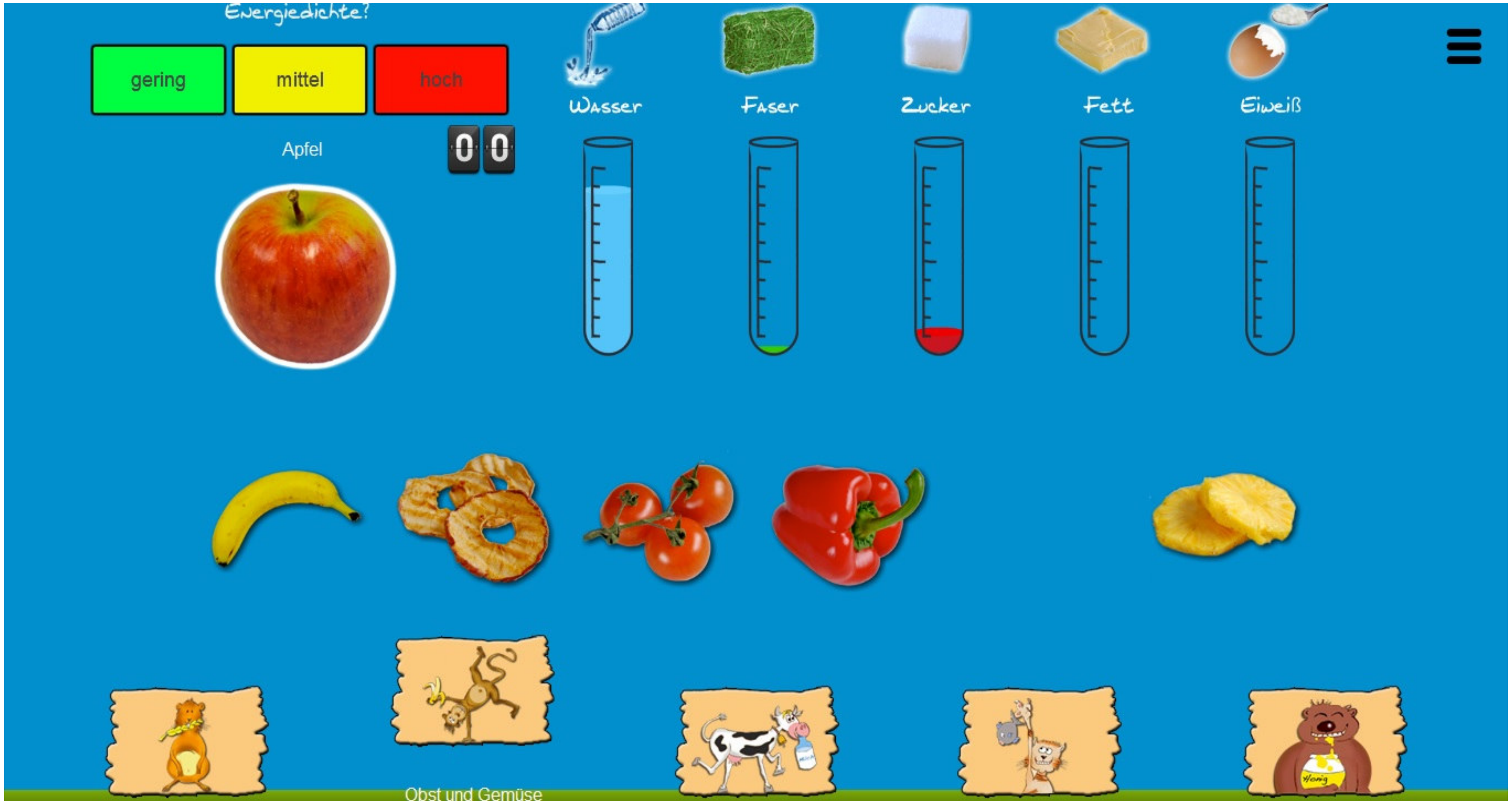

Foods under the microscope (third part after game play): Comparisons and alternatives are discussed

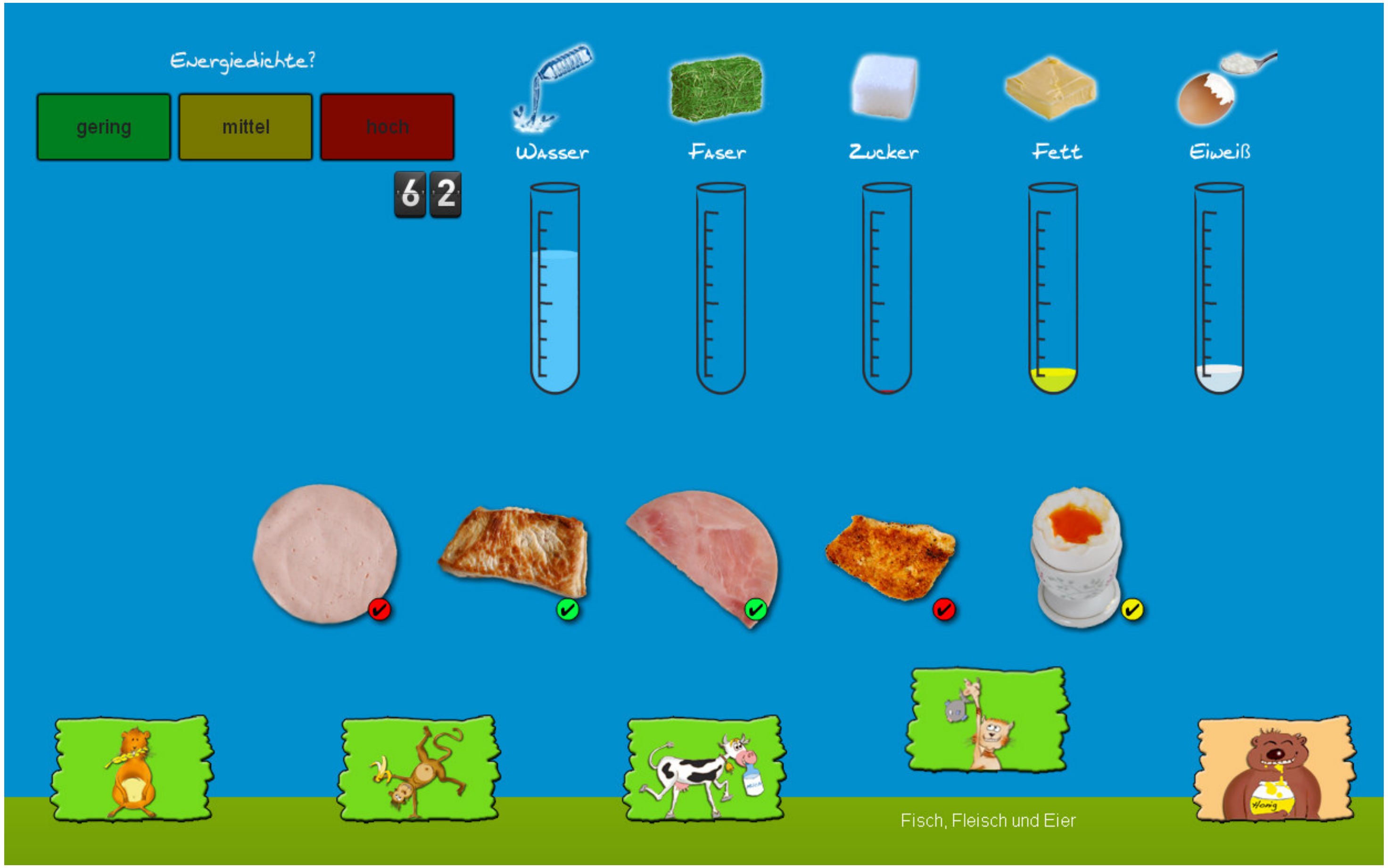

Game module - Liquid rankings on the sugar scale

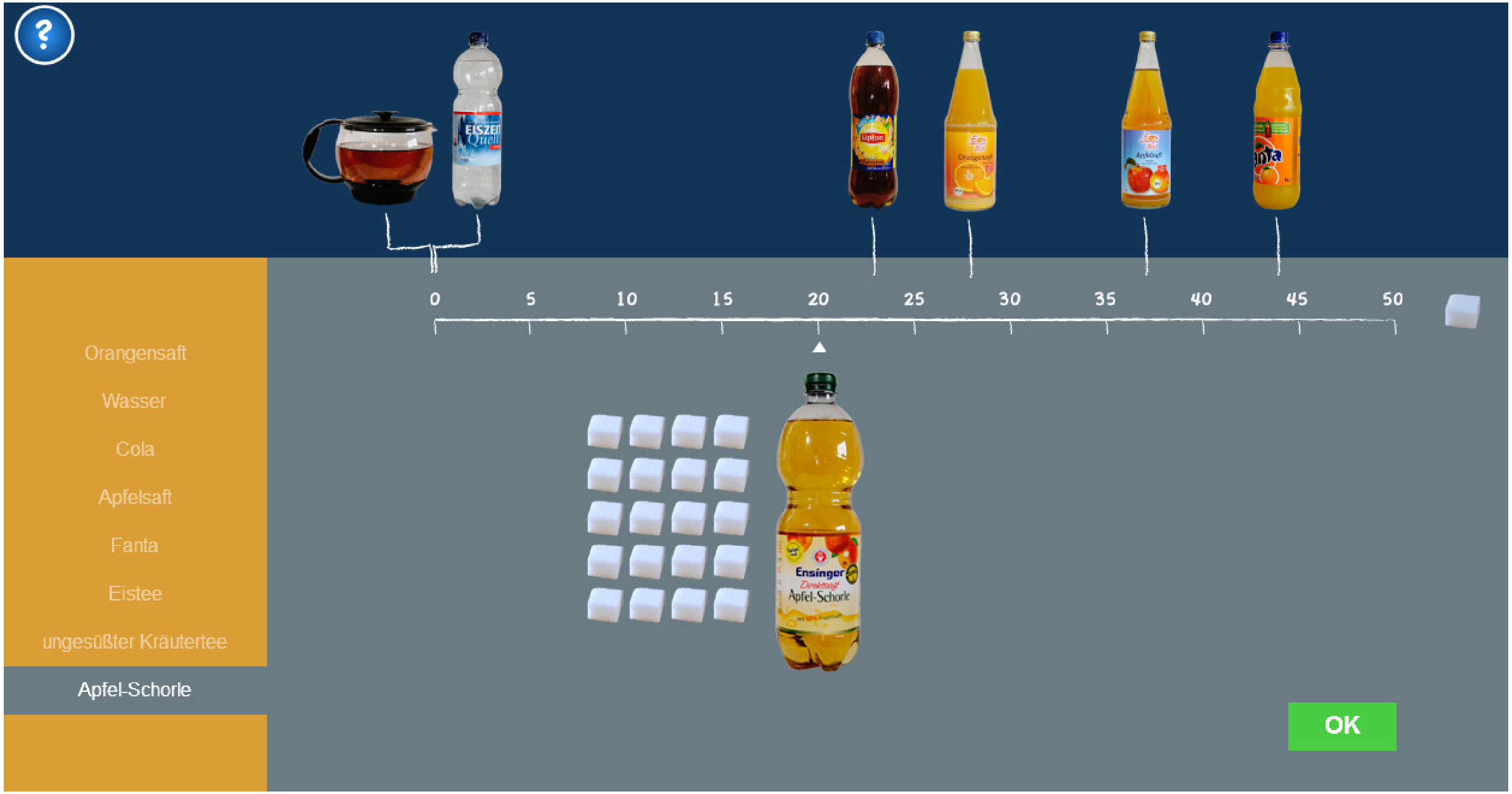

Game module - Kangaroo-Turtle race

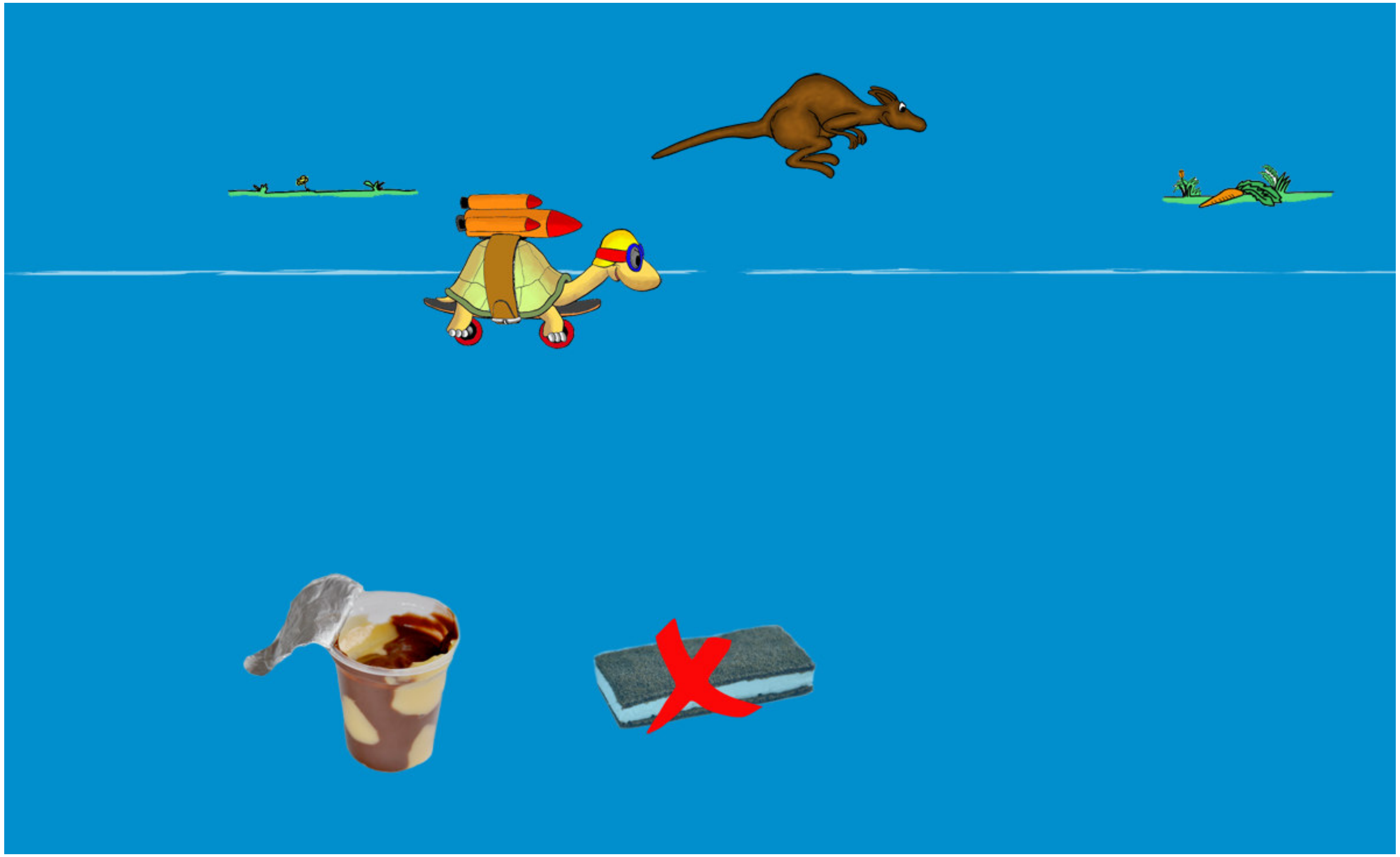

Game module - Bursting bubble game

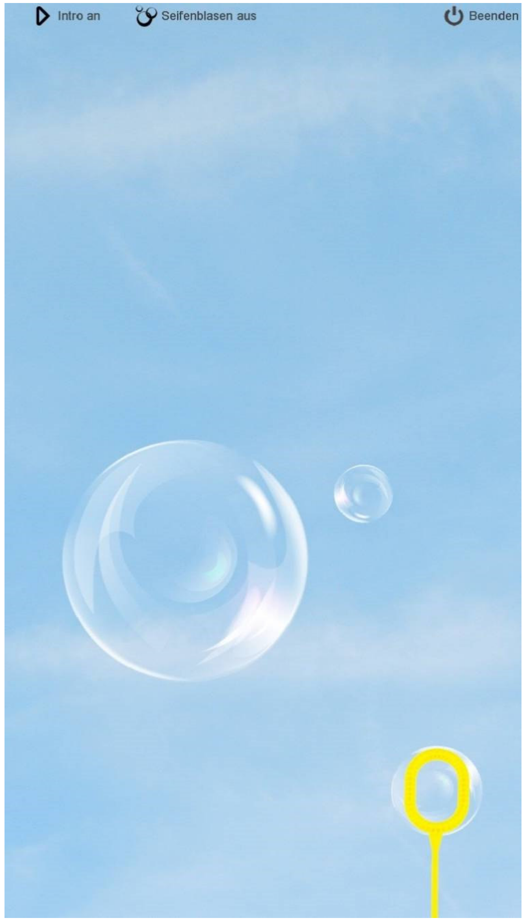

Supplement: Multimedia Appendix 2 [file jmir_v22i4e15725_app2.pdf]
