# Supplementary material for: The Kids Obesity Prevention Program: Cluster Randomized Controlled Trial to Evaluate a Serious Game for the Prevention and Treatment of Childhood Obesity
Source: J Med Internet Res. 2020 Apr 24;22(4):e15725. doi: 10.2196/15725 (PMC7210499; doi:10.2196/15725)
Supplement: Multimedia Appendix 4 [file jmir_v22i4e15725_app4.pdf]

Supplement: Knowledge questionnaire

This questionnaire has been specifically tailored according to the topics addressed in the game (food pyramid, energy density, liquids, eustress and distress, coping with stress). The questionnaire had previously been evaluated in a pilot study. It consists of 13 questions. The answers for the items were transformed to scales ranging from 0 to 100. Subsequently sum scores were calculated and divided by the number of items resulting again in values between 0 and 100. The total knowledge test score includes all items, the dietary energy density score includes the items 3,5,7,9, the food pyramid score the items 1,2,4 and the stress score the items 10,11,12,13. The questionnaire is displayed at the end of the table.

1) Please write down the names of 6 large food groups.

Correct answers: Fruit&Vegetables, Potato&Grains, Milk&Dairy products, Meat&Fish&Eggs, Sweets&Fats, Liquids.

After this first question was answered the sheet of paper was removed before the next sheets of paper were handed out.

2) Assign the indicated food items according to their food groups (food groups provided). Connect them by a line as indicated in the example.

Sugar	
Bread	
Banana	
Carrot	Fruits&Vegetables
Yoghurt	
Ham	Potatoes&Grains
Chocolate	
Sweet Corn	Milk and dairy products
Rice	
Noodles	Meat&Fish&Eggs
Butter	
Salmon	Sweets&Fats
Cheese	
Apple	
Zucchini	

Correct answers: Fruit&Vegetables: banana, carrot, apple, zucchini; Potato&Grains: bread, sweet corn, rice, noodles; Milk&Dairy products: yoghurt; Meat&Fish&Eggs: ham, salmon; Sweets&Fats: chocolate, butter.

3) Please put a cross for the appropriate answer. If a food item has a high energy density (red food item), what does it indicate…

□ that it does not contain much energy per gram (a)
□ that it contains moderate energy per gram (b)
□ that it contains a lot of energy per gram (c)

Correct answer: c)

4) From which food groups should we eat plenty, moderately or sparingly? Please put a cross for every food group accordingly.

Potato&Grains (a):	□ plenty	□ moderately	□ sparingly
Milk&Dairy products (b):	□ plenty	□ moderately	□ sparingly
Meat&Fish&Eggs (c):	□ plenty	□ moderately	□ sparingly
Fruits&Vegetables (d):	□ plenty	□ moderately	□ sparingly
Sweets&Fats (e):	□ plenty	□ moderately	□ sparingly

Correct answers: plenty- Fruit&Vegetables, Potato&Grains, moderately- Milk&Dairy products and Meat&Fish&Eggs, sparingly- Sweets&Fats

5) Please put a cross to indicate the correct answer. In a food item with low energy density (green food item) there is

□ lots of water (a)
□ lots of fat and sugar (b)

Correct answer: a

6) What should you drink to quench your thirst? Please put one cross.

□ fruit juice/nectar (a)
□ lemonade (including coke and fanta; b)
□ water and herbal infusion without sugar (c)
□ apple spritzer or spritzer of other juices (d)
□ lemonade without sugar (e.g. coke light; e)

Correct answer: c

7) Which food items are low in energy density (green food item). Please put 5 crosses.

□ apple (a) □ chocolate (b) □ cucumber (c)
□ ham (d) □ sausage (e) □ mountain cheese (f)
□ chocolate pudding without cream (g) □ potatoes (h)

Correct answers: a, c, d, g, h.

8) How long would you need to play soccer to burn the energy of the following beverages? Please put <u>one</u> cross for <u>a, b and c</u>

a) 1l coke	□ 0 min	□ 30 min	□ 1 h	□ 2 h
b) 1l fruit juice	□ 0 min	□ 30 min	□ 1 h	□ 2 h
c) 1l water	□ 0 min	□ 30 min	□ 1 h	□ 2 h

Corrects answers: a: 2h, b: 2h, c: 0min

9) Please put a circle around the food which is lower in energy density for every food pair as indicated in the example.

e.g. french fries — cooked potatoes
a) sausage — ham
b) dried apple rings — apple
c) chocolate pudding — Milchschnitte (two soft chocolate biscuit layers, in between a sweet creamy layer based on milk and honey)
d) wholemeal bread — white bread
e) banana — chocolate

Correct answers: a – ham, b – apple, c – chocolate pudding, d – wholemeal bread, e – banana

10) Give an example of a situation with healthy stress.

Answer: Several possibilities, e.g. soccer match, theatre performance

11) How can people sometimes realize that they have been exposed to stress over a longer period of time? Please put 3 crosses.

□ stomach ache (a)
□ headache (b)
□ knee ache (c)
□ being nervous/unbalanced (d)
□ good mood (e)
□ having lots of energy (f)

Correct answers: a, b, d

After this section the sheets of paper were removed and the last sheet of paper was distributed.

12) What is an example of a situation with unhealthy stress? Please put one cross.

□ if you feel overwhelmed on a regular basis and are afraid of making mistakes (a)
□ if you are playing soccer with friends (b)
□ if you are excited before a performance or competition (c)

The correct answer is a)

13) What can you do to prevent stress building up? Put 4 crosses.

□ expecting oneself to get many tasks done
□ take breaks (a)
□ I can do nothing to prevent it (b)
□ go outside (c)
□ do physical activities (d)
□ play games (not computer games ; e)
□ eat a lot (f)

Correct answers: b, d, e, f
