# Supplementary material for: The Kids Obesity Prevention Program: Cluster Randomized Controlled Trial to Evaluate a Serious Game for the Prevention and Treatment of Childhood Obesity
Source: J Med Internet Res. 2020 Apr 24;22(4):e15725. doi: 10.2196/15725 (PMC7210499; doi:10.2196/15725)
Supplement: Multimedia Appendix 5 [file jmir_v22i4e15725_app5.pdf]

Supplement: Score Changes from baseline

	IG<br>Mean<br>(95% CI)	CG<br>Mean<br>(95% CI)
Knowledge		
%Total score<br>(Primary outcome)	16 (12)<br>(12-19)	3 (12)<br>(1-6)
% Food pyramid score	28 (16)<br>(22-33)	1 (13)<br>(1-9)
% DED score	14 (2)<br>(7-20)	1 (23)<br>(1-13)
% Stress score	12 (2)<br>(6-18)	0 (22)<br>(1-20)
Physical activity level		
Physical activity level reported by parents	Score: -1.2 (2.8)<br>(-2.0 - -0.3)<br>Activity change:<br>decrease: 37.5 %<br>no change: 50 %<br>increase: 12.5 %	Score: -1.8 (2.9)<br>(-2.7 - -0.9)<br>Activity change:<br>decrease: 38.1%<br>no change: 52.4 %<br>increase: 9.5 %
Physical activity level reported by children	Score: -1.1 (2.4)<br>(-1.9 - -0.3)<br>Activity change:<br>decrease: 30 %<br>no change: 2.5 %<br>increase: 7.5 %	Score: -1.2 (2.6)<br>(-1.9 - -0.4)<br>Activity change:<br>decrease: 26.2 %<br>no change: 61.9 %<br>increase: 11.9 %
Dietary intake pattern		
Index for healthy nutrition reported by parents	Score: 0.8 (2.6)<br>(0-1.6)<br>Change direction:<br>unfavourable 10 %<br>no change: 62.5 %<br>favourable: 27.5%	Score: 0.3 (1.7)<br>(-0.3-0.8)<br>Change direction:<br>unfavourable 11.9 %<br>no change: 73.8 %<br>favourable: 14.3 %
Index for healthy nutrition reported by children	Score: 0.6 (2.6)<br>(-0.2-1.5)<br>Change direction:<br>unfavourable 10 %<br>no change: 67.5 %<br>favourable: 22.5 %	Score: 0.4 (2.2)<br>(-0.3-1.1)<br>Change direction:<br>unfavourable 7.1 %<br>no change: 73.8 %<br>favourable: 19 %
Media consumption		
Watching TV or video films per day reported by parents	Change:<br>decrease: 17.5 %<br>no change: 65 %<br>increase: 17.5 %	Change:<br>decrease: 14.3 %<br>no change: 66.7 %<br>increase: 19 %
Doing gaming activities at a computer per day reported by parents	Change:<br>decrease: 2.5 %<br>no change: 92.5 %<br>increase: 5 %	Change:<br>decrease: 0 %<br>no change: 90.5 %<br>increase: 9.5 %

IG=Intervention group
CG=Control group
CI=Confidence interval
DED=Dietary Energy Density
